# *KRAS* oncogene repression in colon cancer cell lines by G-quadruplex binding indolo[3,2-*c*]quinolines

**DOI:** 10.1038/srep09696

**Published:** 2015-04-08

**Authors:** João Lavrado, Hugo Brito, Pedro M. Borralho, Stephan A. Ohnmacht, Nam-Soon Kim, Clara Leitão, Sílvia Pisco, Mekala Gunaratnam, Cecília M. P. Rodrigues, Rui Moreira, Stephen Neidle, Alexandra Paulo

**Affiliations:** 1 Medicinal Chemistry Group, Research Institute for Medicines (iMed.ULisboa), Faculty of Pharmacy, Universidade de Lisboa, Portugal, Av. Prof. Gama Pinto, 1649-003 Lisbon, Portugal; 2 Cell Function and Therapeutic Targeting Group, Research Institute for Medicines (iMed.ULisboa), Faculty of Pharmacy, Universidade de Lisboa, Portugal, Av. Prof. Gama Pinto, 1649-003 Lisbon, Portugal; 3 The School of Pharmacy, University College London. 29/39 Brunswick Square, London WC1N 1AX, United Kingdom; 4 Medical Genomics Research Center, Korea Research Institute of Bioscience and Biotechnology, Daejeon, 305-333, Republic of Korea

## Abstract

*KRAS* is one of the most frequently mutated oncogenes in human cancer, yet remaining undruggable. To explore a new therapeutic strategy, a library of 5-methyl-indolo[3,2-c]quinoline derivatives (IQc) with a range of alkyldiamine side chains was designed to target DNA and RNA G-quadruplexes (G4) in the promoter and 5′-UTR mRNA of the *KRAS* gene. Biophysical experiments showed that di-substituted IQc compounds are potent and selective *KRAS* G4 stabilizers. They preferentially inhibit the proliferation of *KRAS* mutant cancer cell lines (0.22 < IC_50_ < 4.80 μM), down-regulate *KRAS* promoter activity in a luciferase reporter assay, and reduce both KRAS mRNA and p21^KRAS^ steady-state levels in mutant KRAS colon cancer cell lines. Additionally, IQcs induce cancer cell death by apoptosis, explained in part by their capacity to repress *KRAS* expression. Overall, the results suggest that targeting mutant KRAS at the gene level with G4 binding small molecules is a promising anticancer strategy.

The *RAS* gene family (*KRAS*, *HRAS* and *NRAS*) encodes for a membrane-bound family of G-proteins (p21*^RAS^*), which serve as switches between the endothelial growth factor receptor (EGFR) and the nucleus, controlling downstream processes that influence cell growth/apoptosis. *KRAS* is one of the most frequently mutated oncogenes in human cancers, in particular in pancreatic, colorectal and lung cancers^1^,^2^. In malignant cells, mutated p21*^KRAS^* remains activated and induce cell proliferation as well as other genetic events leading to loss of tumour suppressor function^2^,^3^. Due to the high incidence of these cancers^4^,^5^, the relevance of the *KRAS* oncogene in cancer maintenance and progression is well recognized^6^ as well as its role in increasing resistance to conventional chemotherapy. Consequently the search for KRAS targeting therapies has long been high on the agenda of cancer therapeutics, although the relative lack of success has led to KRAS being labelled as an undruggable target.

Several approaches to pharmacological inhibition of RAS family oncoproteins, particularly of KRAS, have been reported over the past three decades. These included development of direct inhibitors of KRAS mutated proteins, blocking KRAS membrane association and targeting KRAS downstream effectors or upstream activator EGFR^2^. However, all these strategies have shown very disappointing results in clinical trials. It is now recognized that each mutated KRAS protein activate downstream effector signalling in a context and tissue-specific manner and also that it is involved in a complex and dynamic network that can adapt in response to pharmacological inhibitors^2^,^3^. Thus, targeting KRAS mutated proteins or KRAS effectors requires therapeutic agents tailored for both a specific mutated KRAS and cancer tissue.

Other promising approaches are currently being explored, such as RAS-mediated changes in cell metabolism and RAS gene silencing^2^ by the application of miRNAs or siRNA^7^. We have previously demonstrated that miR-143 reduces KRAS expression, chemo-sensitizes colon cancer cells to 5-fluorouracil^8^ and reduces tumour growth *in vivo* with increased apoptosis and reduced proliferation^9^. However such approaches have in particular delivery problems as well as potential off-target effects.

We report here on an approach to targeting KRAS directly at the gene level with small molecules. The promoter of the human K*RAS* gene contains a nuclease hypersensitive element (NHE) composed of six short guanine (G) tracts, from positions −327 to −296 (relative to the exon intron 1 boundary), which is essential for transcription. Within this sequence, a 32-mer region (32R) can form a four-layer higher order DNA structure, a G-quadruplex (G4), involving the G tracts 1-2-3-5, whereas the 21-mer region (21R) can form a three-layer G4, involving the G tracts 1-2-3-4 (Sup. Inf. Figure S1)^10^,^11^,^12^,^13^. These DNA structures have been previously shown to play an important role in the regulation of *KRAS* expression, in particular, in the repression of transcription^12^,^13^. G4 forming motifs have also been located in the 5′ untranslated region (UTR) of *KRAS* and *NRAS* mRNA and shown to be involved in translation inhibition^14^,^15^.

We have been exploring these higher-order nucleic acid structures as possible novel targets for the therapy of colorectal cancer. Targeting the promoter region rather than expressed proteins has several advantages, including the lower likelihood of point mutations and development of drug resistance^16^. In the past decade an intensive search for small-molecules as potent G4 ligands has led to the identification of a large number with anti-proliferative activity in cells^17^,^18^. However, to the best of our knowledge, only three small-molecule chemical types have been identified as *KRAS*/*HRAS* expression down-regulators: low-membrane permeable porphyrins^12^,^13^,^19^,^20^, of which TMPyP4 is the most studied; guanidino anthratiophenediones^21^ and indolo[3,2-*b*]quinolines (IQb)^22^. The *in vitro* structure-activity studies performed with a small library of IQb compounds has suggested that selectivity for G4 and inter-G4 could be modulated by the number and relative position of basic side chains. Moreover, we have identified two compounds with anti-proliferative activity, selective for human colon cancer cells HCT116 compared to rat hepatocytes and a greater capacity to decrease p21*^KRAS^* levels compared to HSP90 protein^22^.

We have now designed a new series of regioisomers, the indolo[3,2-*c*]quinolines (IQc), and have studied *in vitro* their binding to DNA G4 sequences present in the *KRAS* promoter (KRAS21R and KRAS32R) and the RNA G4 sequences in the 192-mer 5′UTR of *KRAS* mRNA. Based on these results we have selected the three more effective and selective IQc compounds and studied their anti-proliferative activities in a panel of *KRAS*-dependent and independent cancer cells, their effects on the down-regulation of *KRAS* expression and their ability to induce apoptosis in colon cancer cell lines with different phenotypes (HCT116 and SW620). The results strongly suggest that targeting *KRAS* expression with cell-permeable G-quadruplex ligands is a promising approach to eventually identifying a compound effective in colorectal cancer therapy.

## Results

### Design and synthesis of indolo[3,2-*c*]quinolines (IQc)

**Figure 1:**
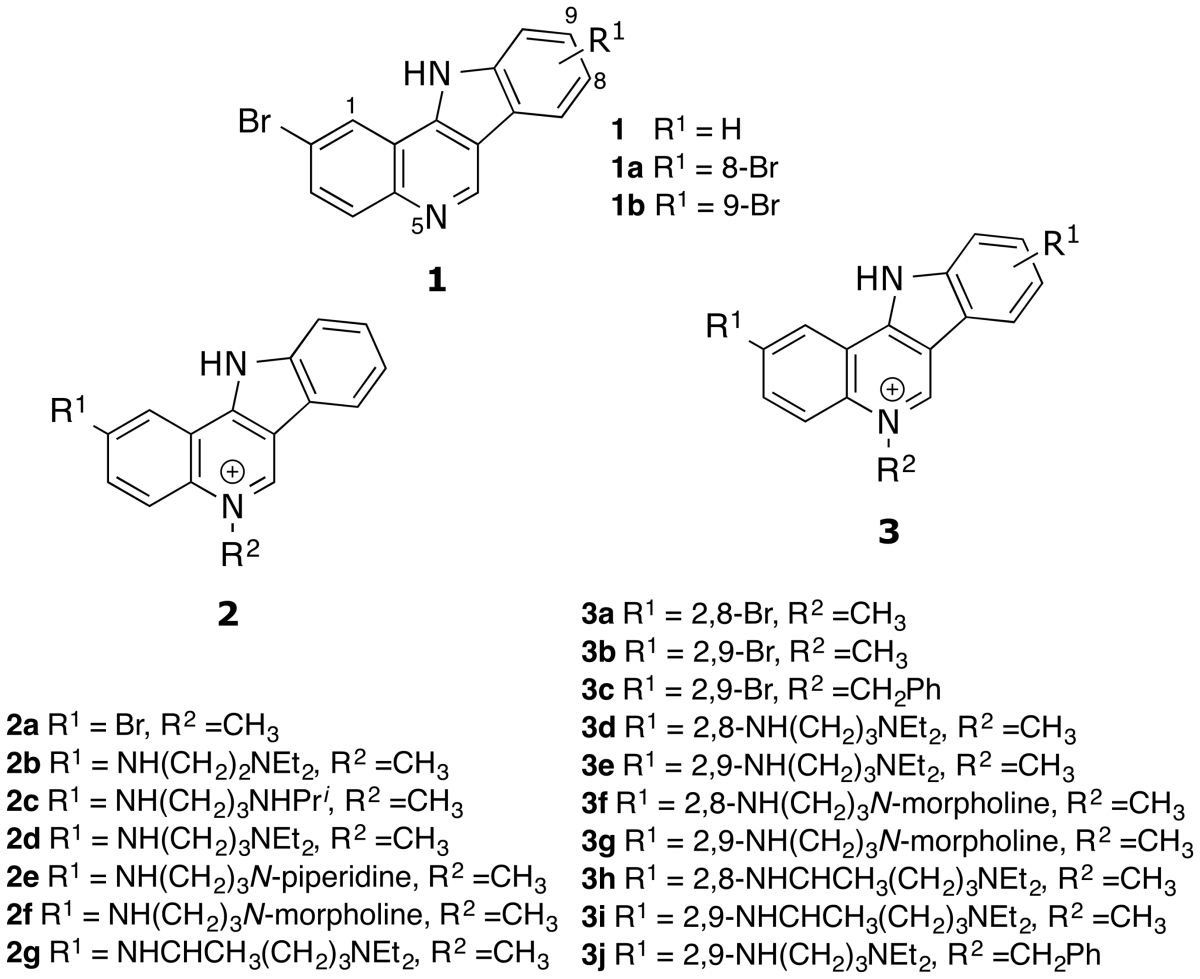
Synthesised indolo[3,2-*c*]quinolines.

Taking advantage of the present knowledge of G4 ligands^18^,^23^ we have designed a series of indolo[3,2-*c*]quinolines (IQcs). Their flat extended heteroaromatic surface can interact with G4s through π-stacking onto terminal G-quartets and the basic side-chains can hydrogen bonding and establish electrostatic interactions with the phosphate backbone and loops/grooves of G4 DNA, either directly or mediated via water molecules (Figure 1). The synthetic pathway to the series is described in detail in the Supplementary Information.

### Selective stabilization of G-quadruplexes by IQc

**Figure 2:**
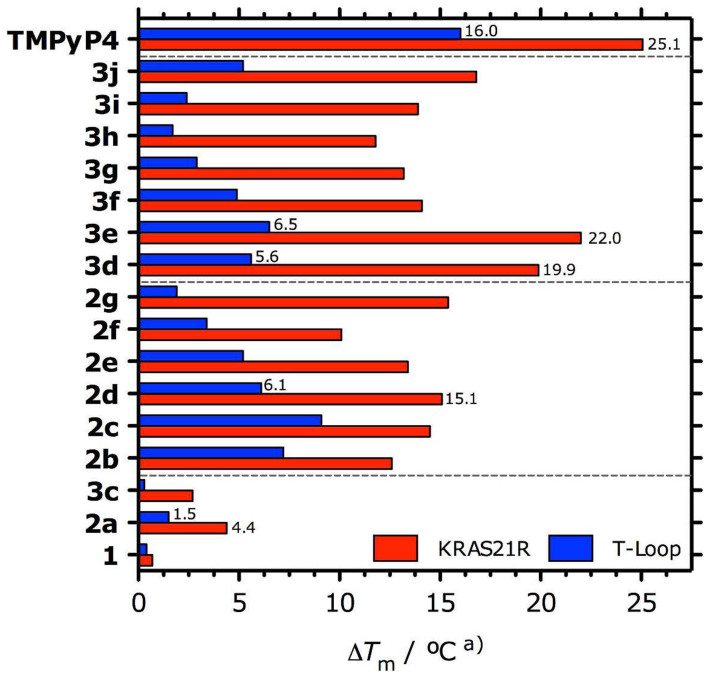
FRET stabilization temperatures (Δ*T_m_*) of KRAS21R G4 and hairpin ds-DNA (T-loop) at 0.2 μM, in K-cacodylate buffer (pH = 7.4, 60 mM K^+^), stabilized by IQc derivatives and TMPyP4 at 1 μM. ^a)^ SD ≤ 0.2°C.

Fluorescence resonance energy transfer (FRET) studies with DNA sequences labelled at the 5′ and 3′ ends, were conducted to evaluate the G4 DNA stabilizing properties of IQcs (at 0.1 to 5 μM ligand concentrations). The G4-forming sequence KRAS21R^11^, together with T-loop, a 26-mer hairpin double-stranded DNA (ds-DNA) sequence were used in the first screening assay. Reproducible IQc dose-dependent melting curves (Sup. Inf.; Figure S3) and stabilization temperature (Δ*T*_m_) values were obtained (Figure 2 and Table S5). Alkyldiamine mono-substituted IQc compounds **2b–g** gave moderate stabilization of the KRAS21R G4 structure, increasing *T*_m_ between 10 and 15°C; the di-substituted alkyldiamine derivatives **3f–j** showed similar behaviour whereas some (**3d, e**) produced greater stabilization with Δ*T*_m_ values varying between 12 and 22°C. IQcs lacking alkyldiamine side chains (**1**, **2a** and **3c**) showed reduced or negligible ability to stabilize the KRAS21R G4. Interestingly, those derivatives with a bulky benzyl N^5^-substituent (**3c** and **3j**) showed decreased KRAS21R G4 stabilization when compared with their mono-substituted counterparts **2a** and **3e**. Moreover, all IQcs showed apparent lower binding affinity to ds-DNA (1.5 < ΔT_m_ < 9.1°C for **2b–g** and 1.6 < ΔT_m_ < 6.5°C for **3d–j**), which suggests selectivity of compounds to G4 compared to ds-DNA.

Two compounds **2d** and **3e** were selected and evaluated for their ability to stabilize the 32-mer sequence in the NHE of the human *KRAS* promoter (KRAS32R)^12^, two G4 motifs in the 5′UTR of *KRAS* mRNA (UTR-1 and UTR-2)^24^ and a 21-mer DNA G4 from the human telomeric sequence (F21T). The KRAS32R sequence has been described as forming two G4 structures which exist in equilibrium. One sequence folds into a G4 (Q_1_) with a *T*_m_ of ~55°C that is disrupted on increasing the temperature and then, the full-length 32-mer sequence folds into a new G4 (Q_2_, *T*_m_ of ~72°C)^13^. In our assay, the KRAS32R sequence (in the presence or absence of IQcs) folded in a single G4 structure (*T_m_* ≈ 54°C), as shown by the single inflection for the first derivative of the FRET melting data (Sup. Inf.; Figure S4). This monophasic behaviour was also observed in the presence of TMPyP4^13^. The results in Table 1 suggest that both **2d** and **3e** are also effective stabilizers of DNA G4 KRAS32R and F21T, but only moderate stabilizers of RNA G4 UTR1 and UTR2. However, the relative stabilizing ability of IQcs was retained for all G4 sequences examined, that is, **3e** is a superior G4-stabilizing ligand than **2d**.

**Figure 3:**
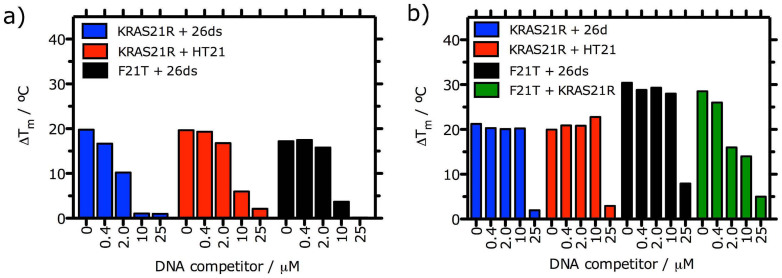
FRET melting competition assay data for (a) 2d and (b) 3e (1 μM) in complex with KRAS21R and F21T G4s (0.2 μM), with increasing concentrations of non-labeled 26ds, HT21 or KRAS21R (0.4 to 25 μM) competitor, in K-cacodylate buffer (pH 7.4, 60 mM K^+^).

To assess selectivity of the IQc compounds **2d** and **3e** to different DNA sequences, we performed competitive FRET experiments with 0.2 μM of labelled KRAS21R and F21T G4 and increasing concentrations of two non-fluorescent competitors, 26mer ds-DNA or the human telomeric sequence (HT21). A decrease in Δ*T*_m_ indicates the displacement of the ligand from labelled G4 by the competitor DNA. Figure 3 shows that **3e** is a more selective G4 ligand than **2d**, with at least 50-fold selectivity for G4 (KRAS21R and F21T) compared with ds-DNA. Interestingly, **3e** also showed selectivity in this assay for KRAS21R over HT21, as it required more than 50-fold higher concentration of HT21 (>10 μM) to displace **3e** from its complex with KRAS21R. To confirm this result we also studied the concentration of KRAS21R required to displace **3e** from its complex with F21T. Figure 3b shows that the induced thermal stability of F21T by **3e** is reduced by 50% in the presence of only 2 μM of competitor KRAS21R. Taken together these results indicate a binding preference of **3e** to bind to KRAS21R than to HT21.

### Characterization of IQc binding to KRAS21R

**Figure 4:**
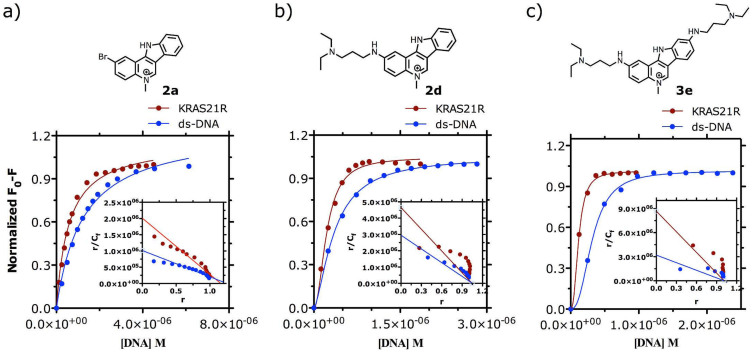
Fluorescence data of (a) 2a, (b) 2d, and (c) 3e (1 μM) in K-cacodylate buffer (pH 7.4, containing 60 mM K^+^) at 25°C, titrated with KRAS21R and HT21 G4s, and 26ds, fitted to the saturation binding equations and Scatchard model (insets).

To characterize the binding of IQc to DNA structures, spectrofluorimetric titration assays were performed with the non-substituted (**2a**), mono-alkyldiamine (**2d**) and di-alkyldiamine (**3e**) derivatives using un-labelled KRAS21R G4 and 26ds-DNA. The emission spectra of compounds **2d** and **3e** with an excitation wavelength (λ_ex_) of 290 nm, were characterized by a broad band centred at 475 nm, while for compound **2a** (λ_ex_ of 275 nm) the emission broad band showed a maximum peak at 465 nm. Their fluorescence intensity was proportional to concentration up to 10 μM. The fluorescence titration spectra of **2a**, **2d**, and **3e** showed 50–70% fluorescence quenching on titration with KRAS21R G4 and 70–95% with 26ds-DNA (Sup. Inf.; Figures S6–S8), thus suggesting the involvement of the aromatic nucleus of IQc compounds in binding to DNA structures. The fluorescence titration data (Figure 4) was fitted to the Scatchard model^25^ (Figure 4, insets). Scatchard plots of **2a** with DNA sequences are close to linearity (Figure 4a), suggesting binding to a single site per G4 or a number of equivalent sites^26^. Further fitting of the fluorescence titration data of **2a** to the one-site saturation binding model gave an association constant (*K_a_*) of 1.7 and 0.8 (×10^6^) M^−1^ for KRAS21R and ds-DNA, respectively (Figure 4a and Table 1). Scatchard plots of **2d** and **3e** (Figure 4b–c, insets) are all concave-down nonlinear curves, which may denote different positive cooperative binding sites in G4, with more than one equivalent and non-independent binding sites^26^. Fitting of the binding data showed no convergence with a two-sites binding model, possibly due to the similarity between the different types of binding and association constants. Therefore, association constants for **2d** and **3e** were obtained from the one-site saturation binding Hill slope model (Figure 4b–c). These are apparent binding constants, i.e. they are macroscopic *K_a_* which reflect the total binding. Calculated *K_a_* values for KRAS21R and ds-DNA range from 2.8 to 8.6 (×10^6^) M^−1^, with Hill constants greater than 1 (Table 1).

Circular dichroism (CD) spectroscopy was performed to examine the likely topology of the KRAS21R G4 and the effect of IQc binding on its conformation. The KRAS21R shows a CD signature for the native folding characteristic of a G4 parallel topology, as previously described^11^. This includes a positive ellipticity maximum around 260 nm and a negative band with a maximum around 240 nm (Sup. Inf.; Figure S9a, solid black line). The titration with **3e** produced only very small CD spectral changes around 260 nm, indicating that G4 topology is maintained.

In order to determine the number of ligand-binding sites, continuous variation analysis (Job plot)^27^ of **3e** with KRAS21R was performed. Plotting of fluorescence as a function of the mole fraction of **3e** (χ) gave linear dependences at high and low molar fractions (Figure S9b). The molar fraction of 0.61 was determined by the intersection of the fluorescence data linear regressions and the results suggest a possible co-existence of **3e**:KRAS21R complexes with stoichiometries of 2:1 and 1:1.

### Anti-proliferative activity of IQc in malignant and non-malignant cell lines

Short-term anti-proliferative assays were carried out by two different assays using a panel of human cancer and non-malignant human cell lines. Concentrations inducing 50% cell growth inhibition (IC_50_) are given in Table 2. Independently of the assay used, all assayed IQc compounds (**2a**, **2d**, **3d**, **3e**), with the exception of **1**, markedly affected the viability of cell lines harbouring mutated *KRAS*, namely the lung cancer cell line A594 (0.40 < IC_50_ < 1.45 μM), the pancreatic cancer cell lines MiaPaCa2 (1.98 < IC_50_ < 2.20 μM) and Panc-1 (0.22 < IC_50_ < 4.80 μM), and the colon cancer cell lines HCT116 (0.14 < IC_50_ < 3.46 μM) and SW620 (0.20 < IC_50_ < 4.74 μM). In comparison, IQcs **2d** and **3e**, but not **2a**, showed a 5–10 fold decrease in effect against the breast carcinoma cell line MCF7 (2.40 < IC_50_ < 11.40 μM), the telomerase-negative human lung fibroblast ALT line (1.93 < IC_50_ < 7.11 μM) and lung fibroblastsWI-38 lines (4.86 < IC_50_ < 10.8 μM). The transformed human embryonic kidney cell line HEK293T (wt *KRAS*) was sensitive to all tested drugs, including TMPyP4, a cationic porphyrin established as a G4 ligand, as well as the anticancer drug 5-fluorouracil (5-FU). Interestingly, TMPyP4 showed a greater cell growth inhibitory effect against HEK293T cells (IC_50_ = 2.97 μM) than to those with mutated *KRAS* (IC_50_(HCT116) = 12.30 μM and IC_50_(SW620) > 20 μM).

### IQc compounds down-regulate *KRAS* gene expression in colon cancer cells

Based on previous results, IQc **2d**, **3d** and **3e** were selected to study effects on those colon cancer cells expressing mutant *KRAS* (HCT116 and metastatic SW620). TMPyP4 and a more membrane-permeable porphyrin with a 14 carbons linear alkyl side chain have previously been shown to bind to G4 forming sequences in the *KRAS* gene promoter region and in the 5′-UTR of *KRAS* mRNA, repressing both gene transcription and translation^12^,^13^,^20^ and so TMPyP4 was included in the experiments as a positive control. To better interpret the relative efficacy of compounds in different cell lines, which may reflect differences arising not only from genetic backgrounds of cell lines but also from differences in compound uptake, compounds were incubated at equitoxic (IC_50_) concentrations.

**Figure 5:**
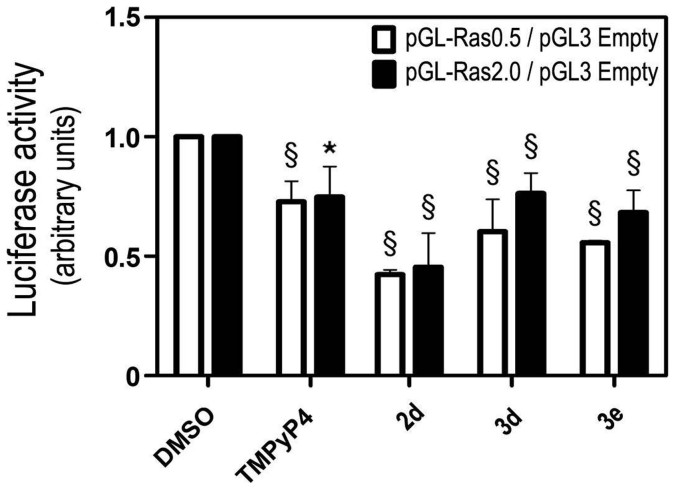
*KRAS* promoter activity is decreased following exposure of HEK293T cells lines to equitoxic (IC_50_) concentrations of compounds TMPyP4, 2d, 3d and 3e. Cells were co-transfected with pGL3-basic vector (empty vector control), or with KRAS promoter luciferase reporter construct PGL-Ras0.5, or PGL-Ras2.0, together with pRL-TK. Twenty-four h later, cells were replated in 96 wells plates, at 5000 cells per well. Subsequently, 24 h after replating, cells were exposed to IC_50_ equitoxic concentration of test compounds IQcs and TMPyP4 and vehicle (DMSO). *KRAS* promoter activity levels were evaluated by Dual-Luciferase assay 72 h after compound exposure. Results are expressed as the luciferase signal ratio of pGL-Ras2.0 or pGL-Ras0.5 to pGL3-basic vector transfected cells, after normalization with Renilla Luciferase. The results are expressed as the mean ± SEM fold-change compared to DMSO exposure, from three independent experiments. *p < 0.05 and § p < 0.01 from DMSO vehicle control.

A first evaluation of the compounds' ability to down-regulate *KRAS* gene transcription by directly acting on the *KRAS* promoter was undertaken with a luciferase reporter assay. For this purpose two different size wild-type promoter constructs were used, containing the G4 sequence and cloned into the Firefly luciferase pGL3 Basic backbone (pGL-Ras0.5, PGL-Ras2.0). These and pGL3 Basic empty (Firefly Luciferase negative control/no promoter) were co-transfected into HEK293T cells together with *Renilla* luciferase pRL-TK (transfection efficiency normalization) as a G4 negative control. This late construct does not harbour G4-forming sequences and is insensitive to G4-related effects/regulation. In general, the data in Figure 5 clearly portraits a reduction of luciferase activity by ~25–50% when compared to *Renilla* basal levels induced by all G4 ligands. Moreover, the same level of promoter activity decrease is induced by compounds in cells transfected with plasmids of different sizes (500 and 2000 bp), suggesting that the target region of tested compounds is at most 500 bp upstream from the start of the coding region, thus coinciding with the region where the G4 sequence is located.

**Figure 6:**
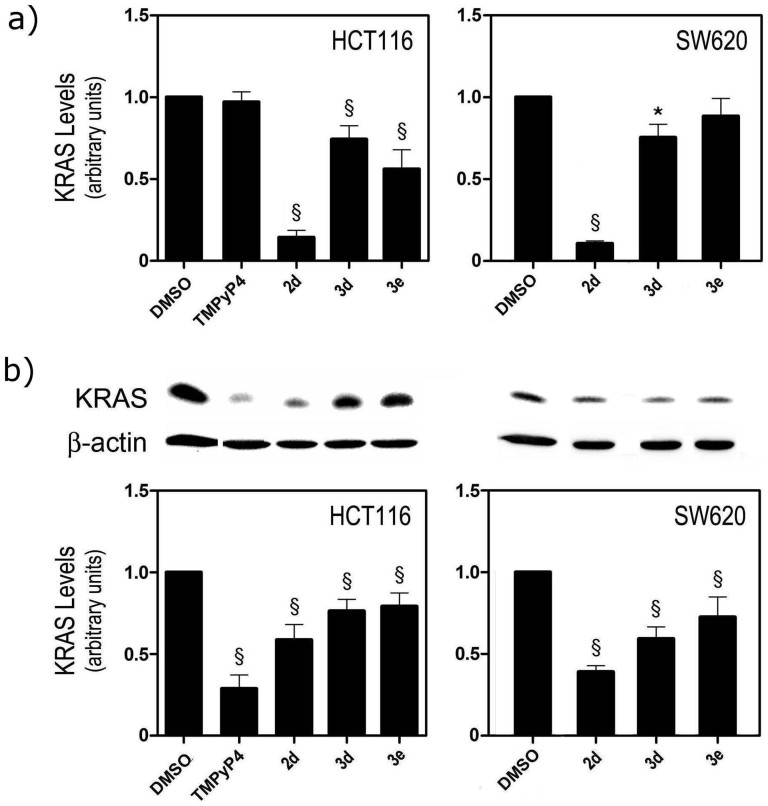
*KRAS* mRNA and protein steady-state expression after exposure of HCT116 and SW620 cells lines to equitoxic (IC_50_) concentrations of compounds TMPyP4, 2d, 3d and 3e for 72 h. (a) *KRAS* mRNA steady-state expression was evaluated by Taqman Real-time RT-PCR using specific Taqman Assays for KRAS and β-Actin for normalization. *KRAS* mRNA steady-state expression levels were calculated by the ΔΔCt method, using DMSO (vehicle control) for calibration; and (b) KRAS protein steady-state expression evaluated by immunoblot relative to control (DMSO vehicle) Results are expressed as mean ± SEM of at least three independent experiments; *p < 0.05 and §*p* < 0.01 from DMSO (vehicle control).

The effects of compounds on *KRAS* transcription in colon cancer cells (HCT116 and SW620) were evaluated by quantifying *KRAS* mRNA steady-state levels using Taqman real-time RT-PCR. The results (Figure 6a) show that IQcs significantly reduced *KRAS* transcription in colon cancer cells. The extent of the effect of **3e** on *KRAS* mRNA steady-state levels depends on cell phenotype, whereas the effects of **2d** and **3d** do not, since these two IQcs were able to reduce *KRAS* mRNA steady-state levels by 90% (**2d**) and 25% (**3d**) in both cell lines. TMPyP4 at 12 μM showed no ability to repress *KRAS* transcription in HCT116 cell line, despite the promoter activity reduction observed in the dual-reporter assay. It must be noted that the effect of TMPyP4 on SW620 cells could not be compared as this cell line showed reduced sensitivity to this compound (IC_50_ > 20 μM).

The capacity of compounds to repress *KRAS* expression was further studied by immunoblotting as test compounds may also inhibit *KRAS* translation by stabilization of RNA G4s in the 5′-UTR of *KRAS*, in particular **3e** (Table 1). p21*^KRAS^* protein levels were significantly reduced (by 20–40%) in both cancer cell lines after treatment with IQc (Figure 6b) and the relative potency of compounds followed the trend **3e** < **3d** < **2d**. In agreement with its reported capacity to accumulate in cytoplasm, bind to UTR1 and UTR2 RNA G4^14^,^20^ and inhibit *NRAS* translation^15^, TMPyP4 reduced the levels of p21*^KRAS^* by ~75% in HCT116 cells.

### Induction of general cell death and apoptosis by IQc compounds

Down-regulation of mutated *KRAS* expression by antisense oligonucleotides in colorectal cancer cells and by a MAZ-binding oligonucleotide decoy in pancreatic cancer cells has been associated with increased apoptosis and cell growth arrest^8^,^28^,^29^. Also, G4 ligands such as TMPyP4, BRACO-19 and pyridostatin have been shown to induce apoptosis in cancer cells^18^,^30^.

**Figure 7:**
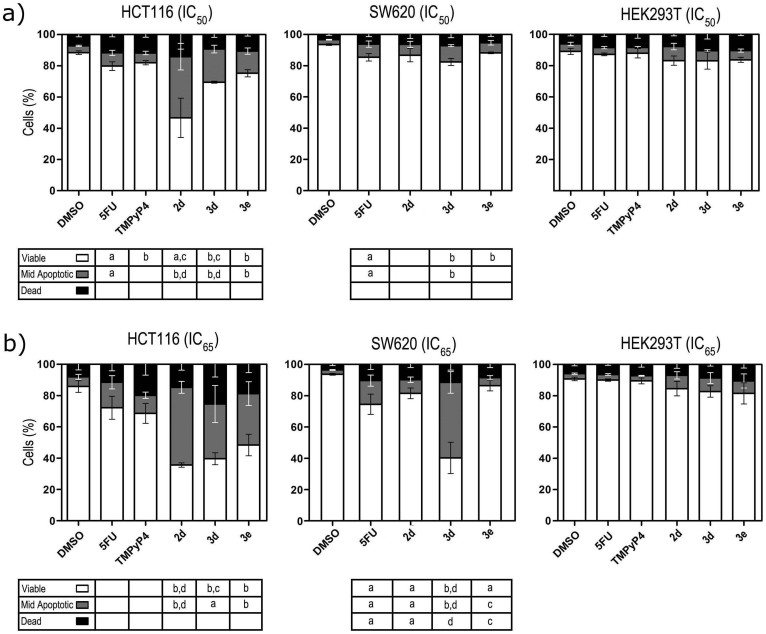
Evaluation of the effect of IQc on cell viability using the ViaCount assay. Cell populations were obtained by Guava ViaCount flow cytometry following 72 h incubation of HCT116 colon cancer, SW620 metastatic colon cancer, and HEK293T embryonic kidney cell lines with 5-FU, TMPyP4, **2d**, **3d** and **3e** at equitoxic (IC_50_ and IC_65_) concentrations, or DMSO (vehicle control). Results are expressed as the mean percentage (%) of viable (white), mid-apoptotic (grey) and dead (black) cells ± SEM, of at least three different experiments, for (a) IC_50_, and for (b) IC_65_ compound concentrations. a, *p* < 0.05; b, *p* < 0.01 from DMSO (vehicle control); c, *p* < 0.05; and d, *p* < 0.01 from 5-FU.

The ability of IQcs to induce cell death in colon cancer cell lines with different genotypes (HCT116 and SW620) and in the non-malignant cell line HEK293T was studied by a Guava Via Count assay and compared with the behaviour of 5-FU and TMPyP4 at equitoxic concentrations (IC_50_ and IC_65_). Results in Figure 7 show dose-dependent induction of cell death, mainly by apoptosis, by all tested compounds in the HCT116 colon cancer cell line. Compounds **2d**, **3d** and **3e** were able to induce cell death at the IC_65_ concentration by 55–70% whereas 5-FU and TMPyP4 showed reduced effects (Figure 7b). These results were confirmed by evaluation of changes in nuclear morphology by Hoechst staining, and were accompanied by ~2–3.5-fold increased steady-state levels of pro-apoptotic p53 protein in HCT116 cells (Figure S11). In SW620 metastatic colon cancer cells, cell death induction was highly dependent on the number and position of side chains in IQc compounds. In this cancer cell line only compound **3d** induced up to 60% cell death (at IC_65_), mainly by apoptosis, followed in potency by 5-FU. In the non-malignant HEK293T cell line none of the tested drugs, IQcs, 5-FU or TMPyP4, caused significant cell death compared to vehicle control.

## Discussion

KRAS signal transduction pathway is a validated cancer target, to which there are no specific targeting agents as yet available in the clinic^1^,^2^. Since there is now good evidence for the presence of DNA and RNA G-quadruplex structures (G4) in the promoter and 5′-UTR mRNA of *KRAS* and their involvement in gene expression downregulation^12^,^13^,^14^, we have exploited these findings and have designed a series of indolo[3,2-c]quinoline derivatives (IQcs; Figure 1) with the aim of explore these nucleic acid structures as possible novel targets for anticancer drug therapy.

The data presented here shows that *in vitro* IQcs are effective stabilizers of the 21-mer and 32-mer *KRAS* G4s, and to a lesser extent of the mRNA *KRAS* G4 UTR-1 and UTR-2, human telomeric G4 and duplex DNA (Figure 2 and Table 1). Structure-activity relationships established on the basis of increased *T_m_* determined by FRET were in agreement with those previously reported for IQb series of compounds^22^. Based on these results compounds **2d** (with one alkyldiamine side chain) and **3e** (with two alkyldiamine side chains) were selected for further *in vitro* studies. **3e** showed greater selectivity (50 fold) than **2d** (2–10 fold) for two DNA G4s (KRAS21R and HT21) compared to ds-DNA. Moreover, the FRET competition experiments showed that **3e** binds preferentially to KRAS21R compared to the human telomeric 21-mer G4 sequence (Figure 3). Competition binding studies have previously shown that some small molecules can bind preferentially to quadruplex compared to triplex or duplex structures^31^,^32^, and/or have differing inductive stabilization effects over G4 DNA sequences of different lengths. This is the case for those IQb compounds with 3 basic side chains in positions 7, 10 and 11, which showed higher Δ*T*_m_ values for 21 mer G4-forming DNA sequences (KRAS21R, HT21, HSP90A) compared to 27 mer G4 DNA (cKit1) or longer G4 DNA structures with more complex topologies (HIF-1α)^22^. We report here that a small molecule can preferentially stabilize one G4 forming sequence over another with the same number of nucleotides. This is undoubtedly due to different folding topologies of KRAS21R and HT21. CD experiments showed that **3e** binds to a parallel G4 formed by the KRAS21R sequence and does not induce any change in topology (Figure S9a), whereas under the same conditions, HT21 folds into a parallel/antiparallel hybrid G4 topology which changes into an antiparallel G4 structure with **3e** binding (Figure S10). Complexes of IQc compounds with KRAS21R were further characterized by spectrofluorimetry. Data for **2d** and **3e** are consistent with the co-existence of 1:1 and 2:1 ligand:G4 complexes as well as an outside stacking binding model. Also, binding affinity constants (*K_a_* ~ 10^6^ M^−1^) are similar to those found for other known G4 end-stacking ligands^33^,^34^.

A comparison has been made of the *in vitro* G4 stabilizing properties with anti-proliferative activity of the IQc compounds with high (**2d, 3d, 3e**) and low (**1, 2a**) G4 Δ*T*_m_ values, against a panel of KRAS-dependent (lung, pancreas and colon) and independent (breast) cancer cell lines and non-malignant cell lines (lung fibroblasts). Only those IQcs with high G4 Δ*T*_m_ values (**2d** and **3e**) showed 5–10 fold superior activity against KRAS-dependent cancer cell lines than against cell lines expressing wt KRAS (MCF7 and lung fibroblasts). IQc compounds **1** and **2a**, without alkylamine side chains and low G4 Δ*T*_m_ values, showed low anti-proliferative activity (**1**) or activity that varied within the cell line panel (**2a**). The anti-proliferative activity found for **2a** may result from its non-selective DNA binding. It has been reported that 2,8-dichloro-5-methyl-indolo[3,2-*c*]quinoline intercalates into ds-DNA^35^, a result that can possibly be extrapolated to **2a** (2-bromo-5-methyl-indolo[3,2-*c*]quinoline).

In colon cancer cell lines with mutated *KRAS*, compound **2d** (one basic side chain) has 5–10 fold higher activity (IC_50_ values of 0.14–0.50 μM) compared to the established anticancer drug 5-FU (2.38–5.39 μM), whereas **3d** and **3e** (two basic side chains) were less effective than **2d**, but equally (**3d**) or more (**3e**) effective than 5-FU and significantly more than TMPyP4. These differences may be due to differing effects on cellular proliferation mechanisms, on cellular uptake and/or intracellular distribution. Assuming a passive diffusion membrane transport for IQcs, compound **2d** is expected to accumulate more inside cells due to its predicted higher lipophilicity than compounds with an additional protonated basic group at physiologic pH (**3d** and **3e**). Similarly, the tetra-cationic porphyrin TMPyP4, which is known to have low cell-membrane permeability, gave higher IC_50_ values. However, the situation may be more complex than this since for example anthratiophenodiones with two basic side chains showed different mechanisms of cell uptake (diffusion or endocytosis) depending on the length of alkyl basic side chains^21^.

In order to take account of the differing abilities of compounds to accumulate inside cells, the mechanism of action was studied at equitoxic concentrations (IC_50_) and TMPyP4 was used as a positive control. Although *in vitro* experiments suggest that TMPyP4 is a poorly selective G4 ligand (Figure 2), it was previously reported that this compound is able to repress *KRAS* transcription. In a dual luciferase assay, TMPyP4 (3–20 μM 24 hours prior to transfection) reduced the mouse *KRAS* promoter activity by 80%^12^ and there was a ca 30% decrease in *KRAS* mRNA levels in Panc-1 cells incubated by 12 or 24 hours with 50 μM of compound^13^. In our reporter assay, ~3 μM TMPyP4 incubated with cells for 72 h after transfection reduced human *KRAS* promoter activity by 25% but did not inhibit *KRAS* transcription in colon cancer HCT116 cells at a 12 μM concentration (Figure 6a). However, at the same concentration TMPyP4 reduced the levels of p21^KRAS^ by >50% in HCT116 cells. These results are in agreement with the reported ability of TMPyP4 to preferentially accumulate in cellular cytoplasm^14^ and inhibit translation. However, it was also shown that this translation inhibitory activity of TMPyP4 is not related to its ability to stabilize the G4 motifs in 5′UTR *KRAS* mRNA but to its non-specific binding affinity to nucleic acids^15^. By comparison, IQcs are shown here to be superior down-regulators of *KRAS* expression. At concentrations <5 μM IQcs **2d**, **3d** and **3e** inhibited *KRAS* promoter activity by up to 50% in the luciferase dual-reporter assay (Figure 5) and inhibited mutated *KRAS* expression at both mRNA and protein levels in the HCT116 and metastatic SW620 colon cancer cell lines (Figure 6). This may be due to a direct effect of IQc compounds on *KRAS* DNA and RNA G4-forming sequences; an indirect effect on transcription and translation factors regulating *KRAS* expression; non-specific binding of compounds to nucleic acids, as observed for TMPyP4; or a combination of these. This is possibly the case of **2d** which showed low selectivity to DNA G4 motifs compared to ds-DNA (Figure 3a) and induced a very large reduction of *KRAS* mRNA levels (to ~10%) that is not accompanied by the same reduction percentage of KRAS protein levels (to ~50%). A direct comparison between compounds in vitro binding strengths to the target G4 KRAS structures and their capacity to inhibit KRAS expression in cells cannot be made for several reasons, including the fact that the mechanism of action was studied using different concentrations for each compound (respective IC_50_) to account for possible differences in cellular uptake.

Having established that IQc compounds repress mutated *KRAS* expression in *KRAS*-dependent colon cancer cells, we then investigated if this would translate into induced cancer cell death by apoptosis. Guanidino anthratiophenediones have been previously shown to stabilize G4 structures *in vitro* in promoter and in 5′-UTR mRNA of *KRAS*, inhibit gene transcription, reduce p21^HRAS^ protein levels, and inhibit proliferation of bladder cancer cells but not through induction of apoptosis^21^. It is shown here that IQc compounds **2d**, **3d** and **3e** were able to induce cell death, mainly by apoptosis, in the wt p53 colon cancer cell line HCT116 in a dose-dependent manner and with a greater effect than 5-FU and TMPyP4 (Figure 7). In this cell line, compounds **2d**, **3d** and **3e** also increased p53 protein levels in agreement with induction of apoptosis. Interestingly, the relative potency of IQcs for inducing cell death (**3e** < **3d** < **2d**) correlates with the induced decrease of p21^KRAS^ protein levels in the same cell line. In the metastatic colon cancer cell line SW620 (mut p53), only 5-FU, TMPyP4 and **3d** induced >20% cell death at the IC_65_ concentration, which does not correlate with the observed relative potency of IQcs to induce p21^KRAS^ decrease in that cell line. P53 status may contribute to these effects, since oncogenic *KRAS* has been shown to sensitize colorectal cancer cells to chemotherapy by wt p53-dependent induction of Noxa^36^. Finally, it must be noted that at concentrations inhibiting 50% cell growth, IQcs did not induce significant cell death in non-malignant cells. It is likely, and consistent with other studies, that G4 ligands are selective for particular driver genes in cancer cells (such as *KRAS*), which have high proliferation rates and thus are physically accessible within chromatin, as has been observed for G4 ligands in cell lines that are dependent on, for example, *cKIT* and *HIF* expression^16^,^37^,^38^,^39^.

This study has shown that IQc derivatives, particularly the di-substituted ones **3d** and **3e**, are effective and selective stabilizers of G4 motifs present in the *KRAS* promoter and the 5′-UTR of *KRAS* mRNA. They are able to down-regulate the expression of the mutated *KRAS* gene through inhibition of transcription and translation, and induce apoptosis accompanied by increased levels of p53 in colon cancer cell lines, at ~1–5 μM concentrations. The higher levels of apoptosis determined by the ViaCount assay and the different cellular response pattern induced by IQc compounds in cancer cells compared to the standard anticancer drug 5-FU and the established G4 ligand TMPyP4, suggest that IQcs, particularly **3d**, which is notable for its apparent ability to induce high levels of apoptosis in metastatic cancer cells, merit further investigation of their mechanism of action, as they may constitute leads for the development of a novel therapeutic agent targeting *KRAS*-dependent cancers. Finally, the results presented here reinforce the more general concept^16^ that targeting genes such as *KRAS* at the gene level in cells that harbor mutated *KRAS* with G4-selective small molecules is a promising anticancer approach.

## Methods

### General Procedure. Synthesis of the mono- and disubtitued dialkylamine indolo[3,2-c]quinolines (2b–g and 3d–j)

Reaction of IQc **2a** or **3a–c** with alkyldiamine, catalysed by Pd(OAc)_2_, in presence of 2-(dicyclohexylphosphino)biphenyl (CyJohnPhos), NaO^t^Bu and solubilized in ^t^BuOH:DME (1:1) was performed in close vessel, under microwave radiation. Purification was performed by preparative thin-layer chromatography (P-TLC) on neutral aluminum oxide. After NMR characterization, compounds were precipitated in their hydrochloric salt form (additional details in Supplementary Information).

### Oligonucleotide sequences

Oligonucleotide sequences were purchased from Eurofins MWG Synthesis GmbH, Germany or STAB VIDA Genomics Lab, Caparica, Lisbon, Portugal. The labelled oligonucleotides used in the FRET assays had attached the donor fluorophore FAM (6-carboxyfluorescein) and the acceptor fluorophore TAMRA (6-carboxytetramethylrhodamine): KRAS21R: 5′-FAM-AGGGCGGTGTGGGAAGAGGGA-TAMRA-3′; KRAS32R: 5′-FAM-AGGGCGGTGTGGGAAGAGGGAAGAGGGGGAGG-TAMRA-3′; UTR-1: 5′-FAM-GCGGCGGCGGAGG-TAMRA-3′; UTR-2: 5′-FAM-UGUGGGAGGGGCGGGUCUGGG-TAMRA-3; T-Loop:5′-FAM-TATAGCTATATTTTTTTATAGCTATA-TAMRA-3′; F21T: 5′-FAM-GGGTTAGGGTAGGGTTAGGG-TAMRA-3′. Non-labelled oligonucleotides were used in FRET competition assays and in the spectroscopic studies: ds26: - d(5′-CAATCGGATCGAATT CGATCCGATTG-3′); KRAS21R: 5′-AGGGCGGTGTGGGAAGAGGGA-3′; HT21: 5′-GGGTTAGGGTAGGGTTAGGG-3′. Each oligonucleotide was initially diluted to a storage solution at 100 μM in nuclease-free water (not DEPC-treated), purchased from Ambion Applied Biosystems UK.

### FRET melting assay

The ability of indolo[3,2-*c*]quinolines to stabilize DNA sequences was investigated using a Fluorescence Resonance Energy Transfer (FRET) assay as reported elsewhere^22^.

### Fluorescence spectroscopy binding studies

Fluorescence data were collected with a λ_ex_ of 290 nm and a λ_em_ around 475 nm for **2d** and **3e**, and a λ_ex_ of 275 nm and a λ_em_ around 460 for **2a**. Test compounds were prepared as 1 mM in HPLC-grade water (10% DMSO) stock solutions. The rest of the dilutions were performed using K-cacodylate buffer (pH = 7.4, containing 60 mM K^+^). The G4 single-strand oligonucleotide sequences were initially diluted from the storage solution (100 μM) with K-cacodylate, to a stock solution at 25 μM. The titration data were obtained by adding aliquots of previously annealed G4 (heating to 95°C for 10 min, followed by slow cooling to RT) to a solution of the indolo[3,2-*c*]quinoline ligand (1 μM) in K-cacocylate buffer at 25°C (additional details in Supplementary Information).

### Circular dichroism assay

CD spectra were recorded with a JASCO 720 spectropolarimeter, with a photomultiplier suitable for the 200–700 nm range. Unless otherwise stated, by circular dichroism (CD) spectra is meant a representation of molar ellipticity ([θ] in deg cm^2^ dmol^−1^) values vs*.* λ ([θ] = 3298.2 × Δε and Δε = differential absorption/(*bC*) where *b* = optical path and *C* = total DNA concentration). All measurements and operations of the spectropolarimeter were computer controlled. The CD spectra shown are the average of three scans, recorded at 25°C, of previously annealed (heating to 95°C for 10 min, followed by slow cooling to RT) 5 μM unlabelled KRAS21R G4 in K-cacodylate buffer (pH 7.4, containing 60 mM K^+^). Test compound was prepared at 1 mM concentration in HPLC-grade water (10% DMSO) stock solution. The rest of the dilutions were performed using K-cacodylate buffer. Titrations of G4 DNA (5 μM) with indolo[3,2-*c*]quinoline **3e** (added aliquots of a 500 μM stock solution in K-cacodylate buffer) were collected between 220 and 320 nm using 10 mm path-length cuvettes. Buffer baseline was subtracted from each spectrum. The following parameters were used for data collection: data pitch 0.5 nm, bandwidth 1 nm, response 2 s and scan speed 100 nm/min.

### Binding Stoichiometry

The binding stoichiometry of indolo[3,2-c]quinoline derivative **3e** with KRAS21R G4 was obtained by spectrophotofluorimetry at 25°C using the Job method of continuous variation^27^. A previously annealed G4 stock solution at 25 μM (heating to 95°C for 10 min, followed by slow cooling to RT) of the non-labelled oligonucleotide sequences KRAS21R in K-cacodylate buffer pH 7.4 (containing 60 mM K^+^) was used. Test compound was prepared at 1 mM concentration in HPLC-grade water (10% DMSO) stock solution. The rest of the dilutions were performed using K-cacodylate buffer. Total concentration of ligand and G4 in the solution was kept constant ([**3e**] + [G4] = 4 μM) as previously described^40^.

### MTS Short-Term Cytotoxicity Assay

HCT116 human colon carcinoma cells, SW620 human colorectal adenocarcinoma and HEK293 T human embryonic kidney cells were grown in Dulbecco's modified Eagle's medium (DMEM) supplemented with 10% fetal bovine serum, and 1% antibiotic/antimycotic (Invitrogen, Grand Island, NY, USA) and maintained at 37°C in a humidified atmosphere of 5% CO_2_. Cells were seeded in 96 well plates at 5.000 cells/well. Twenty-four hours after cell plating, media was removed and replaced with fresh media containing test compounds and 5-FU (Sigma), a common cytotoxic agent used in colon cancer treatmen^8^,^9^,^41^, or vehicle control (DMSO). Following 72 h of compound exposure, cell viability was evaluated using the CellTiter 96 AQueous Non-Radioactive Cell Proliferation Assay (Promega, Madison, WI, USA), using 3-(4,5-dimethylthiazol-2-yl)-5-(3-carboxymethoxyphenyl)-2-(4-sulfophenyl)-2H-tetrazolium, inner salt (MTS) as previously described^42^. Cell viability data were expressed as mean ± SEM or mean ± SD from at least three independent experiments. IC_50_ and IC_65_ values were determined using GaphPad Prism v.5.00 (GraphPad Software). (Performed at Cell Function and Therapeutic Targeting Group, iMed.ULisboa)

### Sulforhodamine B (SRB) Short-Term Cytotoxicity Assay

Human cell lines, breast carcinoma (MCF7), lung carcinoma (A549), pancreatic cancer (MIA PaCa2, Panc-1), immortalised telomerase-negative human lung fibroblast (ALT) and normal human lung fibroblast (WI-38), were all purchased from American Type Cell Culture (ATCC). Cell lines were maintained in appropriate medium supplemented with 10% fetal bovine serum (Invitrogen, UK), 2 mM L-glutamine (Invitrogen, Netherlands), and other components as specified by the suppliers. All cell lines were maintained at 37°C, 5% CO_2_, and routinely passaged. Short-term growth inhibition was measured using the SRB assay as described previously^43^ (additional details described in Supplementary Information).

### Luciferase Reporter Assay

HEK293T cells were seeded in 35 mm plates at 150000 cells per well. 24 h later, cells were transiently co-transfected with pGL3-basic vector (empty vector control), or with *KRAS* promoter luciferase reporter construct pGL-Ras0.5, or pGL-Ras2.0, together with pRL-TK (Promega, Madison, WI, USA). *KRAS* promotor luciferase reporters respectively harbour 500 bp and 2000 bp of the human *KRAS* promotor region, and were previously described elsewhere^44^. pGL3 Basic empty was used as negative control, and pRL-TK simultaneously for transfection efficiency normalization and as a G4 negative control. This construct does not harbour G4 sequences, therefore was considered to be insensitive to G-quadruplex-related effects/regulation^39^. Transfections were performed using Lipofectamine 2000 (Invitrogen), according to the manufacturer's instructions. 24 h after transfection, cells were replated in 96 wells plates, at 5000 cells per well. Subsequently, 24 h after replating, IQc compounds, TMPyP4, and vehicle control (DMSO) were added to the cells at IC_50_ equitoxic concentration. Finally, 72 h after compound incubation, cells were lysed and firefly and *Renilla* luciferase activities were measured using the Dual-Luciferase®Reporter Assay System (Promega). *KRAS* promoter activity levels were expressed as the luciferase signal ratio of pGL-Ras2.0 or pGL-Ras0.5 to pGL3-basic vector transfected cells, after normalization with *Renilla* Luciferase. The results are expressed as the mean ± SEM fold-change compared to DMSO exposure, from three independent experiments.

### Total RNA extraction and Taqman Real-time RT-PCR

HCT116, SW620 and HEK293 T cells were seeded in 35 mm plates at 150000 cells per well. IQc compounds, TMPyP4 and 5-FU and vehicle control (DMSO) were added to the cells 24 h after plating, at IC_50_ equitoxic concentration. After 72 h of compound exposure, cells were collected and processed for total RNA extraction using TRIZOL reagent (Invitrogen) according to the manufacturer's instructions. Samples were homogenized in TRIZOL reagent using a motor-driven Bio-vortexer (No1083; Biospec Products, Bartlesfield, OK) and disposable RNAse/DNAse free sterile pestles (Thermo Fisher Scientific, Inc., Chicago, IL). RNA was quantified using a NanoDropH spectrophotometer, and typically showed A260/280 ratios between 1.9 and 2.1. Evaluation of steady-state expression of KRAS mRNA was performed by Taqman Real-time PCR assay, detailed in Supplementary Information.

### Total protein extraction and immunoblotting

HCT116, SW620 and HEK293 T cells were seeded in 35 mm plates at 150000 cells per well. IQc compounds, TMPyP4, and 5-FU and vehicle control (DMSO) were added to the cells 24 h after plating, at IC_50_ equitoxic concentration. After 72 h of compound exposure, cells were collected and processed for total protein extraction, as previously described^9^ (additional details described in Supplementary Information).

### Guava ViaCount assay

ViaCount assay was used with Guava easyCyte 5HT Flow cytometer (Guava Technologies, Inc., Hayward, CA, USA), as previously described^45^,^46^, to evaluate viable, apoptotic and dead cell populations, on HCT116, SW620 and HEK293T cells exposed to IQc, 5-FU and TMPyP4 at IC_50_ and IC_65_ concentrations, and a vehicle control (DMSO) (additional details described in Supplementary Information).

## Supplementary Material

Supplementary Information Supplementary information

## Figures and Tables

**Table 1:** FRET stabilization temperatures (Δ*T_m_*) of KRAS32R, UTR-1, UTR-2 and F21T G4s (0.2 μM) stabilized by IQc derivatives **2a**, **2d** and **3e** (1 μM). Apparent association constants (*K*_a_) and Hill constants (*h*) of the binding of **2a**, **2d** and **3e** with the *KRAS* G4 (KRAS21R) and with double-stranded DNA (26ds), obtained from spectrofluorimetric titration assays

	Δ*T_m_* (°C)^a^				*K*_a_ × 10^6^ (M^−1^)^b^/*h*^c^	
	KRAS32R	UTR-1	UTR-2	F21T	KRAS21R	26ds
**2a**				5.6	1.7/--	0.8/--
**2d**	10.7	5.1	2.0	17.7	4.4/2.0	2.8/1.5
**3e**	17.0	15.7	9.9	30.7	8.6/2.4	3.2/2.5

a)SD ≤ 0.2°C;

b)SD ≤ 0.2 × 10^6^ M^−1^;

c)SD ≤ 0.3.

**Table 2:** Short term anti-proliferative activity (IC_50_/μM) for IQc (**1**, **2a**, **2d**, **3d**, **3e**), TMPyP4 and 5-fluorouracil (5-FU) using a panel of human cancer^a^ and non-malignant^b^ cell lines

	1	2a	2d	3d	3e	TMPyP4	5-FU
A594c)		0.67 (9.3)^e^	0.40 (12.5)^e^		1.45 (7.0)^e^		
ALTc)		4.75	1.93		7.11		
MCF7c)		2.88	2.40		11.40		
MiaPaCa2c)		2.20			1.98		
Panc1c)		2.75	0.22		4.80		
HCT116d)	9.91	0.14	0.46	3.46	1.80	12.39	2.38
SW620d)	10.66	0.20	0.59	4.74	1.79	>20	5.39
HEK293Td)	8.03	0.30	0.64	6.57	3.12	2.97	2.66
WI-38c)		6.26	4.86		10.08		

a)The panel of cancer cell lines used are: A594 (lung); ALT (telomerase negative human lung fibroblast); MCF7 (breast); MiaPaCa2 (pancreatic); Panc1 (chemo-resistant pancreatic); HCT116 (colon); SW620 (metastatic colon).

b)Non-malignant human cell lines used include: Wi-38 (lung fibroblast); HEK293T (embryonic kidney).

c)Measured by the SRB assay.

d)Measured by the MTS assay.

e)Selectivity index given by: SI = IC_50_(WI-38)/IC_50_(A594).
