# Supplementary material for: KRAS oncogene repression in colon cancer cell lines by G-quadruplex binding indolo[3,2-c]quinolines
Source: Sci Rep. 2015 Apr 8;5:9696. doi: 10.1038/srep09696 (PMC5382548; doi:10.1038/srep09696)
Supplement: Supplementary Information [file srep09696-s1.pdf]

SUPPLEMENTARY INFORMATION

KRAS ONCOGENE REPRESSION IN COLON CANCER CELL LINES BY G-QUADRUPLEX BINDING INDOLO[3,2-*c*]QUINOLINES

João Lavrado[1], Hugo Brito[2], Pedro M. Borralho[2], Stephan A. Ohnmacht[3], Nam-Soon Kim[5], Clara Leitão[3], Sílvia Pisco[3], Mekala Gunaratnam[3], Cecília M. P. Rodrigues[2], Rui Moreira[1], Stephen Neidle[3], Alexandra Paulo[1]*

[1] Medicinal Chemistry Group, Research Institute for Medicines (iMed.ULisboa), Faculty of Pharmacy, Universidade de Lisboa, Portugal, Av. Prof. Gama Pinto, 1649-003 Lisbon, Portugal.

[2] Cell Function and Therapeutic Targeting Group, Research Institute for Medicines (iMed.ULisboa), Faculty of Pharmacy, Universidade de Lisboa, Portugal, Av. Prof. Gama Pinto, 1649-003 Lisbon, Portugal.

[3] The School of Pharmacy, University College London. 29/39 Brunswick Square, London WC1N 1AX, United Kingdom.

[4] Medical Genomics Research Center, Korea Research Institute of Bioscience and Biotechnology, Daejeon, 305-333, Republic of Korea.

Table of Contents

Figure S1 1
Chemistry 1
General 1
Synthesis 2
NOE difference experiments 9
NMR chemical shifts assignment of indolo[3,2-*c*]quinoline derivatives 11
Elemental analysis assays 14
FRET melting assays 14
Fluorescence spectra of saturation binding studies 16
Circular dichroism assay 18
Sulforhodamine B (SRB) short-term cytotoxicity assay 19
Total protein extraction and immunoblotting 19
Total RNA extraction and Taqman real-time RT-PCR 20
Guava ViaCount assay 20
Hoechst Staining 20
Figure S11 21
References 21

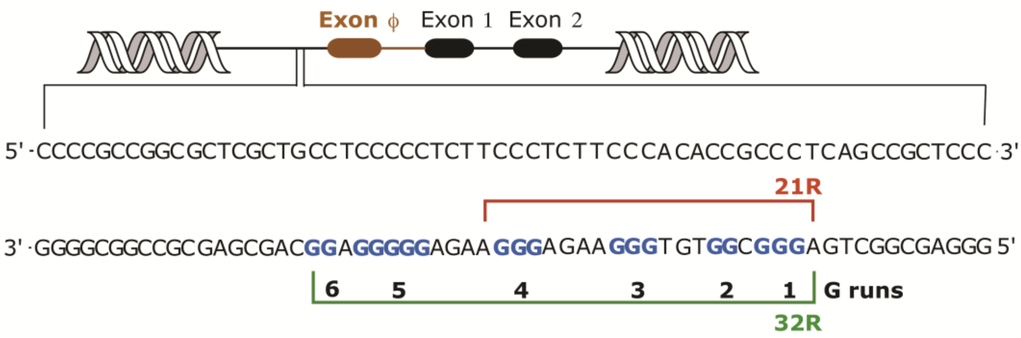

Figure S1 | Schematic representation of the human *KRAS* promoter with its 21-mer (21R) and 32-mer (32R) NHE. The G runs are numbered from 1 to 6 and labelled in blue in the sequence.

Chemistry

General

Chemicals were purchased from Sigma-Aldrich Chemical Co. Ltd., Spain and used without further purification. Microwave reactions were made on a CEM Focused Microwave™ Synthesis System, Model Discover, equipped with the IntelliVent Pressure Control System. All compounds were characterized by NMR spectroscopy, recorded on a Bruker Avance 400 spectrometer at 400 MHz (^{1}H NMR) and 100 MHz (^{13}C NMR), using solvent as internal reference. Chemical shifts (δ) are expressed in ppm. Signal splitting patterns are described as singlet (s), doublet (d), triplet (t), quartet (q), quintet (quint.) and multiplet (m), or combination thereof. Coupling constants (*J*) are given in Hz. The purity of compounds submitted to biological tests were in all cases ≥95% as determined from elemental analysis C, H, and N analysis (Supporting Information), carried out by the Unit for Elemental Analysis, University of Santiago de Compostela, Spain, on a LECO model CHNS-932 elemental analyser. Mass spectra were recorded using a Micromass Quattro Micro API, Watters. Mass spectrum was obtained by direct infusion on "Full Scan" mode (m/z 60-800) and sample ionization was made in positive and negative electrospray ionization mode (ESI+ and ESI-). Melting points (mp) were determined using a Bock-Monoscop M. Reactions were monitored by thin-layer chromatography (TLC) using coated silica gel plates (Merck, aluminium sheets, silica gel 60 F254) and aluminium oxide matrix plates (Sigma-Aldrich, PET support, F254). Preparative thin layer chromatography (PTLC) was performed in neutral aluminium oxide 60 G type E (Merck, 200x200 mm glass support). Fluorescent conjugated oligonucleotides were purchased from Eurofins MWG Operon, Germany. FRET measurements were made on a DNA Engine Opticon (MJ Research) and fluorescence spectroscopy data were collected on a Cary-Eclipse fluorescence spectrophotometer equipped with a Cary Peltier-thermostatted cuvette holder with 1 cm path-length cell. CD spectra were recorded on a JASCO J-720 spectropolarimeter, calibrated with an aqueous solution of 0.06% d-10-(1)-camphor sulfonic acid at 290 nm.

Synthesis

The indolo[3,2-*c*]quinoline (IQc) derivatives **2-3** were prepared from IQc scaffold **1** according to the route depicted in Scheme S1 and starts with the preparation of the 4-chloro-6-bromoquinoline (**9**) by the Gould-Jacobs cyclization.[1] Reaction of the 4-bromoaniline with diethyl-2-(ethoxymethylene)malonate gave **5** which, by thermal cyclization, gave the intermediate ethyl 6-bromo-4-hydroxyquinoline-3-carboxylate (**6**). Hydrolysis of **6** in basic media gave the 6-bromo-4-hydroxyquinoline-3-carboxylic acid (**7**) and subsequent thermal decarboxylation gave **8**. Vilsmayer-Haack reaction of **8** with phosphorus oxychloride gave the final 4-chloro-6-bromoquinoline intermediate (**9**). The synthesis of the IQc nucleus was accomplished by coupling of the intermediate **9** with a 1*H*-benzo[*d*][1,2,3]triazole (**11**) as previously described by Molina *et al*..[2] Reaction of 4-chloro-6-bromoquinoline with commercially available 1*H*-benzo[*d*][1,2,3]triazole (**11a**) or 6-bromo-1*H*-benzo[*d*][1,2,3]triazole (**11b**), obtained from a ultrasound-assisted reaction of 4-bromobenzene-1,2-diamine (**10**) with sodium nitrite, as previously described,[3] gave **12**. Cyclization by thermal decomposition of the 1*H*-benzo[*d*][1,2,3]triazole moiety of **12**, by a Graebe-Ullmann condensation, resulted in the indolo[3,2-*c*]quinolines (**1**). Alkylation of **1** was achieved by microwave-assisted reaction with iodomethane or (iodomethyl)benzene. IQc derivatives **2b-g** and **3d-j** were obtained in the basic form, after treatment with sodium carbonate. Structures of all compounds were established on the basis of 2-D ^{1}H and ^{13}C heterocorrelation NMR experiments (HMQC and HMBC) and their assignements are presented in tables S1-S3. The deshielding effect observed for ^{13}C NMR signals of C2, C8 and C9 of derivatives **2b-g** and **3d-j** corroborate the introduction of the alkyldiamine side-chains and NOE difference experiments (NOE difference experiments Section) confirmed the position of bromide in position 8 or 9 of **3a** and **3b**, respectively. The purity of IQc derivatives **2b-g** and **3d-j** was ≥ 95 %, as hydrochlorides (acid form), determined from elemental analysis (Elemental analysis assays Section).

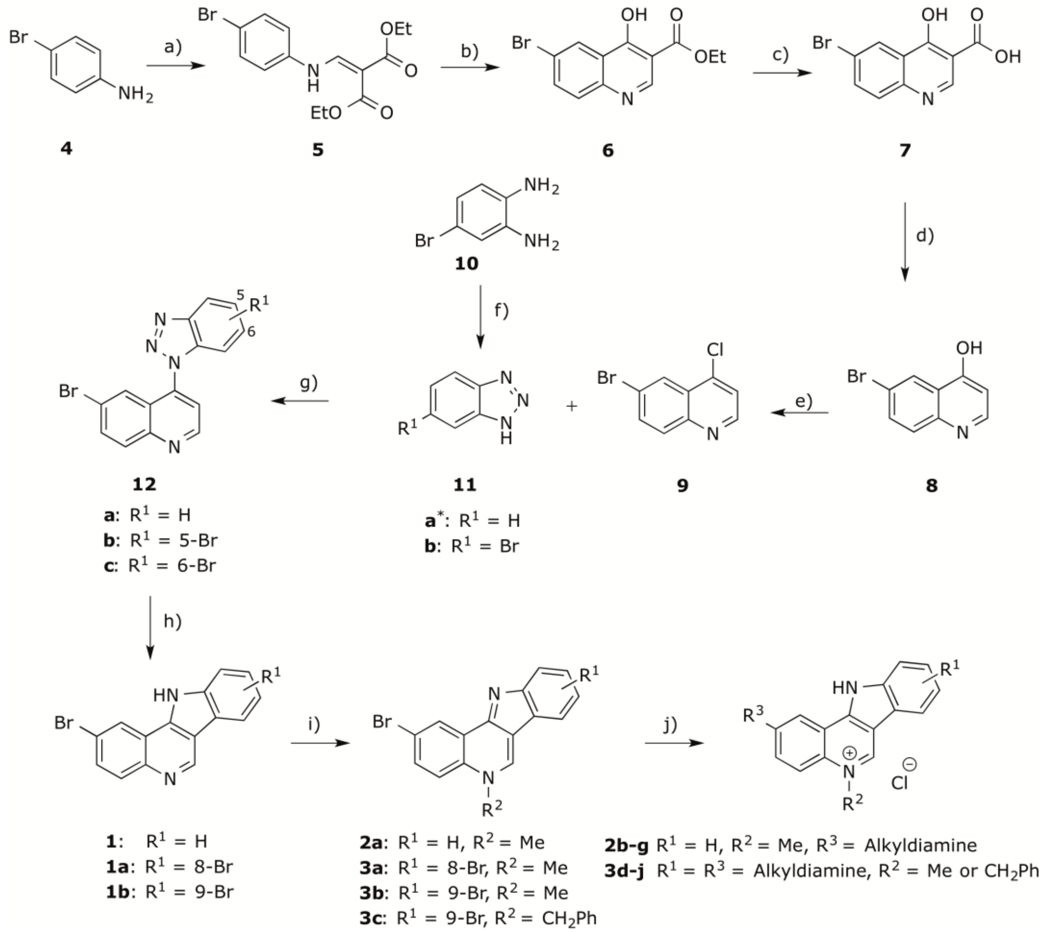

Scheme S1 | Synthetic pathway for the synthesis of the indolo[3,2-*c*]quinoline derivatives. a) diethyl-2-(ethoxymethylene)malonate, reflux; b) diphenyl ether, reflux; c) NaOH 10%, reflux; d) diphenyl ether, reflux; e) $POCl_3$, reflux; f) $NaNO_3$, AcOH, sonicated (35 KHz); g) 120-145 ºC; h) PPA, 145 ºC; i) 1. MeI or $PhCH_2I$, ACN or DMF, 120 ºC or 170 ºC, 300 W, 2. Na_2CO_3 5% ; j) Dialkylamine, $Pd(OAc)_2$, CyJonhPhos, Na^tOBu, DME:^tBuOH (1:1), 170ºC, 300 W. * Commercial available.

Diethyl-2{[(4-bromophenyl)amino]methylene}malonate (5). A solution of 4-bromoaniline (**4**, 10.0 g, 58.0 mmol) in diethyl 2-(ethoxymethylene)malonate (13.8 g, 12.9 mL, 63.8 mmol, 1.1 eq.) was refluxed for 1h . After this period the mixture was cooled until room temperature and added to hexane (300 mL). The precipitate was filtered, washed with hexane and dried to obtain 16.7 g (82%) of pure **5** as a white solid. ^{1}H NMR (400 MHz, DMSO) δ 10.65 (d, *J* = 9.8 Hz, 1H), 8.35 (d, *J* = 10.6 Hz, 1H), 7.61 – 7.47 (m, 2H), 7.36 (d, *J* = 8.9 Hz, 2H), 4.30 – 4.07 (m, 4H), 1.25 (dd, *J* = 12.8, 6.4 Hz, 6H).

Ethyl-6-bromo-4-hydroxyquinoline-3-carboxylate (6). A solution of **5** (6.0 g; 17.5 mmol) in diphenyl ether (60 mL) was refluxed for 1h. After cooling, the reaction mixture was added to hexane (200 mL) and the formed precipitate was filtered, washed with hexane and dried to obtain 3.18 g (61%) of **6** as a white solid. ^{1}H NMR (400 MHz, DMSO) δ 8.59 (s, 1H), 8.23 (d, *J* = 2.1 Hz, 1H), 7.61 (d, *J* = 8.2 Hz, 1H), 7.01 (dd, *J* = 8.2, 2.1 Hz, 1H), 4.23 (q, *J* = 7.1 Hz, 2H), 1.28 (t, *J* = 7.1 Hz, 3H).

6-Bromo-4-hydroxyquinoline-3-carboxylic acid (7). A solution of **6** (3.0 g, 10.1 mmol) in NaOH 10% (40 mL) was refluxed for 1h. After cooling the reaction mixture to 0-5 ºC, the pH was adjusted to 1 with HCl 10 M. The formed precipitate was filtered, washed with water and dried to obtain 2.6 g (96%) of **7** as a white solid. ^{1}H NMR (400 MHz, DMSO)

δ 14.99 (s, 1H), 13.54 (s, 1H), 8.94 (s, 1H), 8.36 (d, J = 2.2 Hz, 1H), 8.04 (dd, J = 8.9, 2.3 Hz, 1H), 7.79 (d, J = 8.9 Hz, 1H).

6-bromoquinolin-4-ol (8). A solution of **7** (2.5 g, 9.3 mmol) in diphenyl ether (30 mL) was refluxed for 1h. The cooled solution was added to hexane (200 mL) and the formed precipitate filtered, washed with hexane and dried to obtain 2.0 g (96%) of **8** as a white solid. ^{1}H NMR (400 MHz, DMSO) δ 11.92 (s, 1H), 8.17 (d, J = 2.4 Hz, 1H), 7.95 (d, J = 7.4 Hz, 1H), 7.79 (dd, J = 8.8, 2.4 Hz, 1H), 7.53 (d, J = 8.8 Hz, 1H), 6.08 (d, J = 7.4 Hz, 1H).

6-bromo-4-chloroquinoline (9). A solution of **8** (2.0 g, 8.9 mmol) in $POCl_3$ (20 mL) was refluxed for 2h. The reaction mixture was cooled, added to water (200 mL) and neutralized with a cold KOH (sat.) solution. The formed precipitate was filtered, washed with water and dried to obtain 2.0 g (93%) of **8** as a light brown solid. ^{1}H NMR (400 MHz, DMSO) δ 8.89 (d, J = 4.7 Hz, 1H), 8.35 (d, J = 2.0 Hz, 1H), 8.05 (d, J = 9.0 Hz, 1H), 8.01 (dd, J = 9.0, 2.0 Hz, 1H), 7.84 (d, J = 4.7 Hz, 1H). ^{13}C NMR (100 MHz, DMSO) δ 151.64, 147.59, 140.56, 134.39, 132.30, 127.26, 126.09, 123.01, 121.96.

6-bromo-1H-benzo[*d*][1,2,3]triazole (11b). A solution of 4-bromo-1,2-diaminobenzene (**10**, 2.0 g, 10.7 mmol) and sodium nitrate (1.84 g, 26.7 mmol, 2.5 eq.) in AcOH (20 mL) was sonicated at 35 KHz for 20 min. After this period the reaction mixture was added to water (250 mL) and extracted with EtOAc (3x200 mL). The combined organic extracts were washed with water (200 mL), dried with brine and anhydrous Na_2SO_4. After solvent removal under reduced pressure, the crude product was purified by recrystallization from boiling EtOH with water to obtain 2.0 g (95%) of **11b** as a light brown solid. mp. 133-135 ºC. ^{1}H NMR (400 MHz, DMSO) δ 15.99 (s broad, NH), 8.29 (s, 1H), 7.99 (d, J = 8.8 Hz, 1H), 7.66 (dd, J = 8.8, 1.6 Hz, 1H). ^{13}C NMR (100 MHz, DMSO) 143.79, 142.05, 130.01, 128,86, 117.88, 117.33. MS (ESI$^+$) $C_6H_4BrN_3$ calculated $[M+H]^+$ 199.96; found $[M+H]^+$ 200.08.

General Procedure S1. 4-(1H-benzo[*d*][1,2,3]triazol-1-yl)-6-bromoquinoline (12a). Homogenized 6-bromo-4-chloroquinoline **9** (1.0 g, 4.1 mmol) and benzotriazole (**11a**, 0.491 g, 4.1 mmol) were heated at 120 ºC for 20 min. The resulting solid was cooled to room temperate and subsequently recrystallized from boiling ethanol with water, to give **12a** as a white solid (1.27 g, 95%). ^{1}H NMR (400 MHz, DMSO) δ 9.23 (d, J = 4.6 Hz, 1H), 8.31 (d, J = 8.4 Hz, 1H), 8.22 (d, J = 9.0 Hz, 1H), 8.08 (dd, J = 9.0, 2.2 Hz, 1H), 8.01 (dd, J = 5.5, 3.4 Hz, 2H), 7.78 (d, J = 8.3 Hz, 1H), 7.71 (ddd, J = 7.9, 5.9, 0.9 Hz, 1H), 7.61 (ddd, J = 8.0, 6.8, 1.0 Hz, 1H). ^{13}C NMR (100 MHz, DMSO) δ 152.28, 148.44, 145.82, 139.12, 134.40, 133.83, 132.31, 129.82, 125.82, 125.78, 124.09, 122.08, 120.37, 118.81, 111.37.

6-Bromo-4-(5-bromo-1H-benzo[*d*][1,2,3]triazol-1-yl)quinoline (12b) and 6-bromo-4-(6-bromo-1H-benzo[*d*][1,2,3]triazol-1-yl)quinoline (12c). A mixture of **9** (2.5 g, 10.3 mmol) and **11b** (2.04 g, 10.3 mmol) was reacted according to general procedure S1. The crude product was purified via flash column chromatography on silica gel using hexane:ethyl acetate (8:2) as eluent, to obtain **12b** (1.44 g, 35%) as a light brown solid and **12c** (2.20 g, 52%) as a white solid. **12b**: ^{1}H NMR (400 MHz, $CDCl_3$) δ 9.17 (d, J = 4.6 Hz, 1H), 8.43 (d, J = 1.2 Hz, 1H), 8.18 (d, J = 9.0 Hz, 1H), 8.00 (d, J = 2.0 Hz, 1H), 7.95 (dd, J = 9.0, 2.1 Hz, 1H), 7.72 (dd, J = 8.8, 1.7 Hz, 1H), 7.62 (d, J = 4.6 Hz, 1H), 7.40 (d, J = 8.8 Hz, 1H). ^{13}C NMR (100 MHz, $CDCl_3$) δ 150.63, 148.71, 147.28, 139.15, 134.56, 132.64, 132.55, 131.86, 125.38, 123.94, 123.33, 122.97, 118.35, 117.53, 111.21. **12c**: ^{1}H NMR (400 MHz, $CDCl_3$) δ 9.17 (d, J = 4.6 Hz, 1H), 8.18 (d, J = 9.0 Hz, 1H), 8.13 (d, J = 8.7 Hz, 1H), 8.03 (d, J = 2.1 Hz, 1H), 7.95 (dd, J = 9.0, 2.1 Hz, 1H), 7.71 (d, J = 1.1 Hz, 1H), 7.65 (dd, J = 8.7, 1.6 Hz, 1H), 7.61 (d, J = 4.6 Hz, 1H). ^{13}C NMR (100 MHz, $CDCl_3$) δ 150.61, 148.75, 144.95, 139.08, 134.64, 134.58, 131.81, 128.96, 125.44, 124.08, 123.80, 123.02, 121.79, 117.39, 112.91.

General Procedure S2. 2-Bromo-11*H*-indolo[3,2-*c*]quinoline (1). A mixture of **12a** (1.27 g,3.9 mmol) in PPA (40.0 g) was heated at 140-150 ºC until the release of N_2 ceased. After 3 hours the reaction mixture was added to cold water (200 mL), neutralized with KOH (sat.) and the precipitate collected by filtration, washed with water and dried under reduced pressure, to give **1a** as a light yellow solid (1,10 g, 95%). ^{1}H NMR (400 MHz, DMSO) δ 12.82 (s, 1H), 9.64 (s, 1H), 8.81 (d, *J* = 2.2 Hz, 1H), 8.34 (d, *J* = 7.8 Hz, 1H), 8.08 (d, *J* = 8.9 Hz, 1H), 7.86 (dd, *J* = 8.9, 2.2 Hz, 1H), 7.74 (d, *J* = 8.2 Hz, 1H), 7.52 (dd, *J* = 8.2, 7.6 Hz 1H), 7.34 (d, *J* = 7.8, 7.6 Hz 1H). ^{13}C NMR (101 MHz, DMSO) δ 145.88, 144.41, 139.28, 139.12, 132.23, 131.32, 126.42, 124.93, 122.05, 121.30, 120.79, 118.93, 118.83, 115.31, 112.48.

2,8-Dibromo-11*H*-indolo[3,2-*c*]quinoline (1b). A mixture of **12b** (1.0 g, 2.5 mmol) and PPA (30 g) was reacted according to General procedure S2 to give **1b** as a light brown solid (0.335 g, 36%). ^{1}H NMR (400 MHz, DMSO) δ 12.91 (s, 1H), 9.64 (s, 1H), 8.76 (s, 1H), 8.59 (s, 1H), 8.06 (d, *J* = 8.8 Hz, 1H), 7.86 (d, *J* = 8.8 Hz, 1H), 7.68 (d, *J* = 8.3 Hz, 1H), 7.62 (d, *J* = 8.3 Hz, 1H). ^{13}C NMR (100 MHz, DMSO) δ 146.25, 144.65, 139.75, 138.02, 132.28, 131.74, 128.85, 125.01, 124.03, 123.47, 119.07, 118.81, 114.54, 114.45, 113.59.

2,9-Dibromo-11*H*-indolo[3,2-*c*]quinoline (1c). A mixture of **12c** (1.0 g, 2.5 mmol) and PPA (40 g) was reacted according to General procedure S2 to give **1c** as a light brown solid (0.487 g, 52%). ^{1}H NMR (400 MHz, DMSO) δ 13.00 (s, 1H), 9.62 (s, 1H), 8.80 (d, *J* = 2.2 Hz, 1H), 8.28 (d, *J* = 8.4 Hz, 1H), 8.06 (d, *J* = 8.9 Hz, 1H), 7.90 (d, *J* = 1.5 Hz, 1H), 7.86 (dd, *J* = 8.9, 2.2 Hz, 1H), 7.49 (dd, *J* = 8.4, 1.5 Hz, 1H). ^{13}C NMR (100 MHz, DMSO) δ 145.87, 144.52, 140.16, 139.56, 132.22, 131.62, 125.03, 124.20, 122.54, 121.20, 119.07, 119.01, 118.77, 115.08, 114.86.

General Procedure S3. 2-Bromo-5-methyl-5*H*-indolo[3,2-*c*]quinoline (2a). A mixture of **1** (0.5 g, 1.7 mmol) and MeI (2.39 g, 1.04 mL, 17.0 mmol) in ACN (5 mL) was stirred, in a close vessel, under microwave radiation (P_{max} = 300 W) at 120 ºC for 2 hours. After this period, the solvent was removed at reduced pressure. The remaining residue was suspended in Na_2CO_3 5% aqueous solution (100 mL) and extracted with CH_2Cl_2 (3x50 mL). Combined organic extracts were dried with brine and anhydrous Na_2SO_4, and the solvent removed under reduced pressure to give **2a** as a dark yellow solid (0.382 g, 76 %). ^{1}H NMR (400 MHz, $CDCl_3$) δ 8.80 (d, *J* = 2.3 Hz, 1H), 7.95 (d, *J* = 8.6 Hz, 1H), 7.78 (s, 1H), 7.77 (d, *J* = 8.9 Hz, 1H), 7.58 (d, *J* = 8.6 Hz, 1H), 7.54 (dd, *J* = 8.8, 2.3 Hz, 1H), 7.31 (dd, *J* = 8.9, 7.5 Hz, 1H), 7.27 (dd, *J* = 8.8, 7.5 Hz, 1H), 3.82 (s, 3H). ^{13}C NMR (100 MHz, $CDCl_3$) δ 154.49, 152.06, 135.19, 133.73, 131.91, 126.84, 126.60, 125.18, 121.92, 120.84, 119.27, 119.02, 118.76, 117.63, 117.29, 42.39.

5-Methyl-2,8-dibromo-5*H*-indolo[3,2-*c*]quinoline (3a). Reaction of **1a** (0.5 g, 1.3 mmol) and MeI (1.88 g, 0.828 mL, 13.0 mmol) in ACN (5 mL) according to General Procedure S3, gave **3b** as a dark yellow solid (0.332 g, 62%). ^{1}H NMR (400 MHz, DMSO) δ 9.53 (s, 1H), 8.86 (d, *J* = 2.2 Hz, 1H), 8.34 (d, *J* = 2.0 Hz, 1H), 8.08 (d, *J* = 9.2 Hz, 1H), 8.03 (dd, *J* = 9.2, 2.2 Hz, 1H), 7.74 (d, *J* = 8.6 Hz, 1H), 7.58 (dd, *J* = 8.6, 2.0 Hz, 1H), 4.28 (s, 3H). ^{13}C NMR (100 MHz, DMSO) δ 152.24, 150.56, 140.90, 134.94, 132.91, 128.94, 127.42, 126.35, 122.98, 122.28, 121.05, 120.11, 119.29, 115.82, 113.25, 43.38.

5-Methyl-2,9-dibromo-5*H*-indolo[3,2-*c*]quinoline (3b). Reaction of **1b** (0.5 g, 1.3 mmol) and MeI (1.88 g, 0.828 mL, 13.0 mmol) in ACN (5 mL) according to General Procedure S3, gave **3b** as a dark orange solid (0.352 g, 68%). ^{1}H NMR (400 MHz, DMSO) δ 9.39 (s, 1H), 8.81 (s, 1H), 8.05 (d, *J* = 8.3 Hz, 1H), 8.01 (d, *J* = 9.0 Hz, 1H), 7.98 (d, *J* = 9.0 Hz, 1H), 7.95 (s, 1H), 7.39 (d, *J* = 8.3 Hz, 1H), 4.23 (s, 3H). ^{13}C NMR (100 MHz,

DMSO) δ 156.18, 152.82, 139.71, 134.84, 132.47, 126.25, 125.09, 123.09, 122.86, 121.72, 121.55, 120.78, 118.91, 118.74, 116.52, 42.94.

5-Benzyl-2,9-dibromo-5*H*-indolo[3,2-*c*]quinoline (3c). A mixture of **1b** (0.15 g, 0.39 mmol) and (iodomethyl)benzene (0.096 g, 0.44 mmol) in ACN (4 mL) was stirred, in a close vessel, under microwave radiation (P_{max} = 300 W) at 150 ºC for 3 hours. After this period, the solvent was removed and reduced pressure and the resulting residue suspended Na_2CO_3 5 % aqueous solution (100 mL) and extracted with CH_2Cl_2 (3x50 mL). The combined organic extracts were concentrated under reduced pressure and purified via flash column chromatography on silica gel using CH_2Cl_2:MeOH as eluent (gradient from 100:0 to 90:10), to give **3c** as a dark yellow solid (0.076 g, 42%). ^{1}H NMR (400 MHz, $CDCl_3$) δ 8.99 (s, 1H), 8.57 (s, 1H), 8.01 (s, 1H), 7.81 (d, *J* = 8.5 Hz, 1H), 7.61 (d, *J* = 9.2 Hz, 1H), 7.58 (d, *J* = 8.5 Hz, 1H), 7.43 (d, *J* = 9.2 Hz, 1H), 7.34 (m, 3H), 7.09 (d, *J* = 4.4 Hz, 2H), 5.68 (s, 2H). ^{13}C NMR (100 MHz, $CDCl_3$) δ 152.91, 152.03, 136.59, 134.27, 133.54, 133.07, 132.76, 129.78, 129.42, 128.73, 127.42, 126.62, 126.05, 122.38, 120.53, 120.01, 119.57, 118.85, 114.36, 58.59.

General Procedure S4. 2-{[2-(Diethylamino)ethyl]amino}-5-methyl-11*H*-indolo[3,2-*c*]quinolin-5-ium chloride (2b). A solution of **2a** (50.0 mg, 0.16 mmol), 2-(dicyclohexylphosphino)biphenyl (CyJohnPhos, 11.2 mg, 0.032 mmol), $Pd(OAc)_2$ (7.2 mg, 0.032 mmol), NaO^tBu (61.5 mg, 0.64 mmol) and N^1,N^1-diethylethane-1,2-diamine (74.4 mg, 89.9 µL, 0.64 mmol) in ^{t}BuOH:DME (1:1, 2 mL) was stirred in a close vessel, under microwave radiation (P_{max} = 300 W) at 170 ºC for 2 hours. After cooling to room temperature, the crude mixture was filtered over Celite® 545, the solid washed with CH_2Cl_2:MeOH (9:1) and the filtrate evaporated under reduced pressure. The residue was then suspended in Na_2CO_3 5% aqueous solution (50 mL) and extracted with CH_2Cl_2 (3x50 mL). The combined organic extracts were dried with brine, anhydrous Na_2SO_4 and concentrated under reduced pressure. Purification by preparative thin-layer chromatography (P-TLC) on neutral aluminum oxide, using CH_2Cl_2:MeOH (9:1) as eluent and desorbent from the aluminum oxide, gave **2b** as a dark yellow solid (29.3 mg, 56%). After NMR characterization, **2b** was precipitated in its salt form, from CH_2Cl_2 (2 mL) with HCl in Et_2O, filtrated and dried under reduced pressure. ^{1}H NMR (400 MHz, $CDCl_3$) δ 7.99 (s, 1H), 7.89 (d, *J* = 8.0 Hz, 1H), 7.74 (s, 1H), 7.73 (d, *J* = 8.2 Hz, 1H), 7.42 (dd, *J* = 8.0, 7.5 Hz, 1H), 7.22 (d, *J* = 8.8 Hz, 1H), 7.17 (dd, *J* = 8.2, 7.5 Hz, 1H), 6.86 (d, *J* = 8.8 Hz, 1H), 3.82 (s, 3H), 3.24 (m, 2H), 2.73 (t, *J* = 5.0 Hz, 2H), 2.59 (q, *J* = 7.0 Hz, 4H), 1.06 (t, *J* = 7.0 Hz, 6H). ^{13}C NMR (100 MHz, $CDCl_3$) δ 147.14, 134.63, 130.20, 128.63, 126.43, 125.21, 122.55, 120.73, 119.61, 119.36, 118.17, 117.87, 115.62, 103.14, 51.93, 47.22, 43.36, 41.75, 12.44.

2-{[3-(isopropylamino)propyl]amino}-5-methyl-11*H*-indolo[3,2-*c*]quinolin-5-ium chloride (2c). Reaction of **2a** (50.0 mg, 0.16 mmol), CyJohnPhos (11.2 mg, 0.032 mmol), $Pd(OAc)_2$ (7.2 mg, 0.032 mmol), NaO^tBu (61.5 mg, 0.64 mmol) and N^1-isopropylpropane-1,3-diamine (74.4 mg, 89.6 µL, 0.64 mmol) in ^{t}BuOH:DME (1:1, 2 mL), followed by purification according to General Procedure S4 gave **2c** as a dark yellow solid (23.0 mg, 41%). ^{1}H NMR (400 MHz, $CDCl_3$) δ 7.95 (s, 1H), 7.93 (d, *J* = 7.5 Hz, 1H), 7.76 (d, *J* = 7.7 Hz, 1H), 7.72 (s, 1H), 7.45 (dd, *J* = 7.7, 7.5 Hz, 1H), 7.21 (d, *J* = 8.1 Hz, 1H), 7.19 (dd, *J* = 7.7, 7.2 Hz, 1H), 6.83 (d, *J* = 8.1 Hz, 1H), 3.80 (s, 3H), 3.30 (t, *J* = 6.6 Hz, 2H), 2.83 (dd, *J* = 12.5, 6.2 Hz, 1H), 2.77 (t, *J* = 6.5 Hz, 2H), 1.86 (m, 2H), 1.10 (t, *J* = 6.2 Hz, 6H). ^{13}C NMR (100 MHz, $CDCl_3$) δ 152.21, 151.65, 146.44, 133.74, 127.97, 125.73, 124.98, 122.37, 119.99, 118.93, 118.40, 118.03, 117.20, 115.27, 102.08, 48.89, 45.79, 43.13, 42.54, 29.47, 22.97.

2-{[3-(Diethylamino)propyl]amino}-5-methyl-11*H*-indolo[3,2-*c*]quinolin-5-ium chloride (2d). A solution of **2a** (50.0 mg, 0.16 mmol), CyJohnPhos (11.2 mg, 0.032 mmol), $Pd(OAc)_2$ (7.2 mg, 0.032 mmol), NaO^tBu (61.5 mg, 0.64 mmol) and N^1,N^1-

diethylpropane-1,3-diamine (83.3 mg, 100.9 µL, 0.64 mmol) in ^{t}BuOH:DME (1:1, 2 mL) was reacted and purified according to General Procedure S4 to give **2d** as a dark yellow solid (16.2 mg, 28%). ^{1}H NMR (400 MHz, CDCl$_3$) δ 8.02 (s, 1H), 7.94 (d, *J* = 7.6 Hz, 1H), 7.78 (s, 1H), 7.77 (d, *J* = 7.4, 1H), 7.45 (dd, *J* = 7.6, 7.4 Hz, 1H), 7.26 (d, *J* = 9.1 Hz, 1H), 7.20 (dd, *J* = 7.4, 7.1 Hz, 1H), 6.85 (d, *J* = 9.1 Hz, 1H), 3.83 (s, 3H), 3.32 (t, *J* = 6.0 Hz, 2H), 2.58 (m, 6H), 1.85 (t, *J* = 6.7 Hz, 2H), , 1.08 (t, *J* = 7.1 Hz, 6H). ^{13}C NMR (100 MHz, CDCl$_3$) δ 152.45, 151.67, 146.69, 133.79, 127.95, 125.69, 125.04, 122.55, 119.96, 118.94, 118.34, 118.07, 117.22, 115.33, 102.18, 52.24, 46.92, 43.91, 42.55, 25.92, 11.84.

5-Methyl-2-{[3-(piperidin-1-yl)propyl]amino}-11*H*-indolo[3,2-*c*]quinolin-5-ium chloride (2e). A solution of **2a** (50.0 mg, 0.16 mmol), CyJohnPhos (11.2 mg, 0.032 mmol), Pd(OAc)$_2$ (7.2 mg, 0.032 mmol), NaO^{t}Bu (61.5 mg, 0.64 mmol) and *3*-(piperidin-1-yl)propan-1-amine (91.4 mg, 102.1 µL, 0.64 mmol) in ^{t}BuOH:DME (1:1, 2 mL) was reacted and purified according to General Procedure S4 to give **2e** as a dark yellow solid (28.8 mg, 48%). ^{1}H NMR (400 MHz, CDCl$_3$) δ 8.31 (s, 1H), 7.77 (d, *J* = 9.1 Hz, 1H), 7.75 (d, *J* = 8.3 Hz, 1H), 7.53 (s, 1H), 7.37 (dd, *J* = 9.1, 7.6 Hz, 1H), 7.29 (d, *J* = 8.5 Hz, 1H), 7.16 (dd, *J* = 8.3, 7.6 Hz, 1H), 6.82 (d, *J* = 8.5 Hz, 1H), 4.03 (s, 3H), 3.26 (m, 2H), 2.50 (t, *J* = 6.4 Hz, 2H), 2.46 (m, 4H), 1.86 (t, *J* = 6.0 Hz, 2H), 1.66 (m, 4H), 1.51 (m, 2H). ^{13}C NMR (100 MHz, CDCl$_3$) δ 147.32, 134.78, 127.78, 126.37, 123.32, 120.93, 120.50, 119.58, 119.31, 117.57, 115.89, 114.06, 101.06, 58.15, 54.82, 43.65, 43.35, 26.37, 25.40, 24.61.

5-Methyl-2-[(3-morpholinopropyl)amino]-11*H*-indolo[3,2-*c*]quinolin-5-ium chloride (2f). Reaction of **2a** (50.0 mg, 0.16 mmol), CyJohnPhos (11.2 mg, 0.032 mmol), Pd(OAc)$_2$ (7.2 mg, 0.032 mmol), NaO^{t}Bu (61.5 mg, 0.64 mmol) and 3-morpholinopropan-1-amine (92.2 mg, 93.5µL, 0.64 mmol) in ^{t}BuOH:DME (1:1, 2 mL), followed by purification according to General Procedure S4 gave **2f** as a dark yellow solid (29.5 mg, 49%). ^{1}H NMR (400 MHz, CDCl$_3$) δ 7.97 (s, 1H), 7.96 (d, *J* = 7.7 Hz, 1H), 7.80 (d, *J* = 1.9 Hz, 1H), 7.78 (d, *J* = 8.1 Hz, 1H), 7.47 (dd, *J* = 7.7, 7.6 Hz, 1H), 7.26 (d, *J* = 8.7 Hz, 1H), 7.23 (dd, *J* = 8.1, 7.6 Hz, 1H), 6.87 (dd, *J* = 8.7, 1.9 Hz, 1H), 3.81 (s, 3H), 3.76 (m, 4H), 3.33 (t, *J* = 6.0 Hz, 2H), 2.52 (t, *J* = 6.5 Hz, 2H), 2.50 (m, 4H), 1.86 (2t, *J* = 6.5, 6.0 Hz 2H).^{13}C NMR (100 MHz, CDCl$_3$) δ 154.14, 153.01, 146.93, 134.23, 128.66, 126.25, 125.88, 123.42, 120.47, 119.47, 119.04, 118.89, 117.80, 116.21, 102.91, 67.75, 58.24, 54.47, 44.12, 43.12, 26.01.

2-{[5-(Diethylamino)pentan-2-yl]amino}-5-methyl-11*H*-indolo[3,2-*c*]quinolin-5-ium chloride (2g). A solution of **2a** (50.0 mg, 0.16 mmol), CyJohnPhos (11.2 mg, 0.032 mmol), Pd(OAc)$_2$ (7.2 mg, 0.032 mmol), NaO^{t}Bu (61.5 mg, 0.64 mmol) and N^1,N^1-diethylpentane-1,4-diamine (101.3 mg, 124.0 µL, 0.64 mmol) in ^{t}BuOH:DME (1:1, 2 mL) was reacted and purified according to General Procedure S4 to give **2g** as a dark yellow solid (30.1 mg, 48%). ^{1}H NMR (400 MHz, CDCl$_3$) δ 8.15 (s, 1H), 7.91 (d, J = 7.9 Hz, 1H), 7.86 (s, 1H), 7.79 (d, J = 7.6 Hz, 1H), 7.42 (dd, J = 7.9, 7.4 Hz, 1H), 7.30 (d, J = 9.2 Hz, 1H), 7.18 (dd, J = 7.6, 7.4 Hz, 1H), 6.86 (d, J = 9.2 Hz, 1H), 3.90 (s, 3H), 3.72 (m, Hz, 1H), 2.53 (q, J = 7.1 Hz, 4H), 2.42 (t, J = 6.5 Hz, 2H), 1.59 (m, 4H), 1.25 (d, J = 6.8 Hz, 3H), 1.02 (t, J = 7.1 Hz, 6H). ^{13}C NMR (100 MHz, CDCl$_3$) δ 152.05, 151.04, 145.84, 134.11, 127.90, 125.75, 124.90, 122.46, 120.11, 119.01, 118.41, 117.86, 117.48, 115.25, 103.01, 52.76, 48.46, 46.74, 42.60, 35.04, 23.72, 20.65, 11.51.

2,8-bis{[3-(diethylamino)propyl]amino}-5-methyl-11*H*-indolo[3,2-*c*]quinolin-5-ium chloride (3d). A solution of **3a** (50.0 mg, 0.13 mmol), CyJohnPhos (9.1 mg, 0.026 mmol), Pd(OAc)$_2$ (5.8 mg, 0.026 mmol), NaO^{t}Bu (99.9 mg, 1.04 mmol) and N^1,N^1-diethylpropane-1,3-diamine (135.4 mg, 163.8 µL, 1.04 mmol) in ^{t}BuOH:DME (1:1, 2 mL) was reacted and purified according to General Procedure S4 to give **3d** as a dark orange solid (12.3 mg, 20%). ^{1}H NMR (400 MHz, CDCl$_3$) δ 8.23 (s, 1H), 7.76 (s, 1H), 7.55 (d, J = 7.0 Hz, 1H), 7.36 (d, J = 8.6 Hz, 1H), 7.02 (s, 1H), 6.86 (d, J = 8.6 Hz, 1H), 6.50 (d, J =

8.0 Hz, 1H), 4.09 (s, 3H), 3.36 (m, 2H), 3.28 (t, J = 6.2 Hz, 2H), 2.58 (m, 12H), 1.85 (m, 4H), 1.08 (dt, J = 6.8, 6.2 Hz, 12H). ^{13}C NMR (100 MHz, $CDCl_3$) δ 152.17, 151.95, 149.33, 147.50, 133.78, 128.37, 125.59, 122.46, 120.44, 120.11, 117.98, 115.65, 110.48, 102.05, 99.23, 53.01, 52.84, 47.61, 47.58, 44.70, 44,63, 43.99, 26.90, 26.50, 12.63, 12.60.

2,9-bis{[3-(diethylamino)propyl]amino}-5-methyl-11*H*-indolo[3,2-*c*]quinolin-5-ium chloride (3e). A solution of **3b** (50.0 mg, 0.13 mmol), CyJohnPhos (9.1 mg, 0.026 mmol), $Pd(OAc)_2$ (5.8 mg, 0.026 mmol), NaO^tBu (99.9 mg, 1.04 mmol) and N^1,N^1-diethylpropane-1,3-diamine (135.4 mg, 163.8 µL, 1.04 mmol) in ^{t}BuOH:DME (1:1, 2 mL) was reacted and purified according to General Procedure S4 to give **3e** as a dark orange solid (15.8 mg, 25%). ^{1}H NMR (400 MHz, $CDCl_3$) δ 7.94 (s, 1H), 7.64 (s, 1H), 7.45 (d, J = 8.0 Hz, 1H), 7.22 (d, J = 8.2 Hz, 1H), 7.00 (s, 1H), 6.79 (d, J = 8.2 Hz, 1H), 6.50 (d, J = 8.0 Hz, 1H), 4.04 (s, 3H), 3.39 (m, 2H), 3.37 (m, 2H), 2.69 (m, 12H), 1.99 (m, 4H), 1.24 (dt, J = 6.5 Hz, 12H). ^{13}C NMR (100 MHz, $CDCl_3$) δ 152.96, 151.47, 149.18, 147.22, 133.07, 128.33, 124.09, 121.25, 120.30, 119.54, 117.84, 115.95, 110.20, 102.23, 97.64, 52.87, 52.80, 47.60, 47.55, 44.74, 44.45, 43.67, 26.91, 26.62, 12.62, 12.55.

5-Methyl-2,8-bis[(3-morpholinopropyl)amino]-11*H*-indolo[3,2-*c*]quinolin-5-ium chloride (3f). A solution of **3a** (50.0 mg, 0.13 mmol), CyJohnPhos (9.1 mg, 0.026 mmol), $Pd(OAc)_2$ (5.8 mg, 0.026 mmol), NaO^tBu (99.9 mg, 1.04 mmol) and 3-morpholinopropan-1-amine (149.9 mg, 151.9 µL, 1.04 mmol) in ^{t}BuOH:DME (1:1, 2 mL) was reacted and purified according to General Procedure S4 to give **3f** as a dark orange solid (17.3 mg, 25%). ^{1}H NMR (400 MHz, CDCl3) δ 8.05 (s, 1H), 7.83 (s, 1H), 7.60 (d, J = 7.8 Hz, 1H), 7.36 (d, J = 8.6 Hz, 1H), 7.14 (s, 1H), 6.92 (d, J = 8.6 Hz, 1H), 6.57 (t, J = 7.3 Hz, 1H), 4.09 (s, 3H), 3.85 (m, 8H), 3.45 (m, 4H), 2.64 (m, 12H), 2.03 (m, 4H). ^{13}C NMR (100 MHz, $CDCl_3$) δ 151.76, 151.32, 148.90, 146.69, 132.64, 128.69, 121.75, 120.20, 119,40, 117.74, 115.51, 110.00, 101.95, 101.56, 67.78, 67.73, 58.38, 58.23, 54.50, 54.48, 44.44, 44.11, 43.22, 26.25, 26.03.

5-Methyl-2,9-bis[(3-morpholinopropyl)amino]-11*H*-indolo[3,2-*c*]quinolin-5-ium chloride (3g). A solution of **3b** (50.0 mg, 0.13 mmol), CyJohnPhos (9.1 mg, 0.026 mmol), $Pd(OAc)_2$ (5.8 mg, 0.026 mmol), NaO^tBu (99.9 mg, 1.04 mmol) and 3-morpholinopropan-1-amine (149.9 mg, 151.9 µL, 1.04 mmol) in ^{t}BuOH:DME (1:1, 2 mL) was reacted and purified according to General Procedure S4 to give **3g** as a dark orange solid (29.8 mg, 45%). ^{1}H NMR (400 MHz, $CDCl_3$) δ 8.10 (s, 1H), 7.78 (s, 1H), 7.56 (d, J = 8.2 Hz, 1H), 7.35 (d, J = 9.0 Hz, 1H), 7.07 (s, 1H), 6.87 (d, J = 9.0 Hz, 1H), 6.55 (d, J = 8.2 Hz, 1H), 4.04 (s, 3H), 3.74 (m, 8H), 3.31 (m, 4H), 2.51 (m, 12H), 1.87 (m, 4H). ^{13}C NMR (100 MHz, $CDCl_3$) δ 153.93, 150.46, 148.22, 146.34, 133.87, 128.02, 125.17, 122.05, 121,80, 119.59, 118.54, 117.08, 114.75, 109.21, 102.14, 101.24, 67,10, 67.07, 57.72, 57.51, 53.80, 53.77, 43.34, 43,26, 42.50, 25.49, 25.29.

2,8-Bis{[5-(diethylamino)pentan-2-yl]amino}-5-methyl-11*H*-indolo[3,2-*c*]quinolin-5-ium chloride (3h). A solution of **3a** (50.0 mg, 0.13 mmol), CyJohnPhos (9.1 mg, 0.026 mmol), $Pd(OAc)_2$ (5.8 mg, 0.026 mmol), NaO^tBu (99.9 mg, 1.04 mmol) and N^1,N^1-diethylpentane-1,4-diamine (164.6 mg, 201.4 µL, 1.04 mmol) in ^{t}BuOH:DME (1:1, 2 mL) was reacted and purified according to General Procedure S4 to give **3h** as a dark orange solid (11.2 mg, 16%). ^{1}H NMR (400 MHz, $CDCl_3$) δ 8.09 (s, 1H), 7.84 (s, 1H), 7.58 (d, J = 8.0 Hz, 1H), 7.37 (d, J = 8.7 Hz, 1H), 7.09 (s, 1H), 6.90 (d, J = 8.7 Hz, 1H), 6.53 (d, J = 7.7 Hz, 1H), 4.13 (s, 3H), 3.86 (m, 1H), 3.73 (d, J = 4.8 Hz, 1H), 2.66 (q, J = 6.2 Hz, 8H), 2.58 (m, 4H), 1.75 (s, 8H), 1.43 (d, J = 4.8 Hz, 6H), 1.20 (m, 12H). ^{13}C NMR (100 MHz, $CDCl_3$) δ 152.59, 151.94, 147.94, 146.48, 132.96, 128.47, 124.00, 122.11, 120.34, 119.36, 117.94, 115.34, 110.44, 103.51, 100.05, 53.67, 49.53, 49.19, 47.46, 47.42, 43.50, 35.82, 35.77, 24.45, 24.41, 21.54, 21.45, 12.34, 12.26.

2,9-Bis{[5-(diethylamino)pentan-2-yl]amino}-5-methyl-11*H*-indolo[3,2-*c*]quinolin-5-ium chloride (3i). A solution of **3b** (50.0 mg, 0.13 mmol), CyJohnPhos (9.1 mg, 0.026 mmol), $Pd(OAc)_2$ (5.8 mg, 0.026 mmol), NaO^tBu (99.9 mg, 1.04 mmol) and N^1,N^1-diethylpentane-1,4-diamine (164.4 mg, 201.4 µL, 1.04 mmol) in ^{t}BuOH:DME (1:1, 2 mL) was reacted and purified according to General Procedure S4 to give **3i** as a dark orange solid (15.8 mg, 22%). ^{1}H NMR (400 MHz, $CDCl_3$) δ 8.00 (s, 1H), 7.84 (s, 1H), 7.53 (d, *J* = 7.8 Hz, 1H), 7.34 (d, *J* = 8.6 Hz, 1H), 7.11 (s, 1H), 6.86 (d, *J* = 8.6 Hz, 1H), 6.52 (d, *J* = 7.8 Hz, 1H), 4.12 (s, 3H), 3.86 (m, 1H), 3.75 (m, 1H), 2.67 (dq, *J* = 6.9, 6.7 Hz, 8H), 2.57 (m, 4H), 1.76 (m, 8H), 1.44 (d, *J* = 6.6, 6H), 1.20 (dt, *J* = 6.9, 6.8 Hz, 12H). ^{13}C NMR (100 MHz, $CDCl_3$) δ 153.09, 152.06, 148.19, 146.51, 133.16, 128.16, 124.33, 121.95, 120.02, 118.45, 116.23, 115.31, 114.09, 109.96, 103.51, 102.60, 99.45, 53.72, 53.68, 50.10, 49.18, 47.44, 47.40, 44.12, 35.84, 35.59, 24.52, 24.41, 21.62, 21.38, 12.26, 12.11.

5-Benzyl-2,9-bis{[3-(diethylamino)propyl]amino}-11*H*-indolo[3,2-*c*]quinolin-5-ium chloride (3j). Reaction of **3c** (50.0 mg, 0.11 mmol), CyJohnPhos (7.5 mg, 0.021 mmol), $Pd(OAc)_2$ (4.8 mg, 0.021 mmol), NaO^tBu (82.4 mg, 0.85 mmol) and N^1,N^1-diethylpropane-1,3-diamine (110.7 mg, 134.0 µL, 0.85 mmol) in ^{t}BuOH:DME (1:1, 2 mL), followed by purification according to General Procedure S4 gave **3j** as an orange solid (23.7 mg, 39%). ^{1}H NMR (400 MHz, $CDCl_3$) δ 8.86 (s, 1H), 7.78 (s, 1H), 7.60 (d, *J* = 8.1 Hz, 1H), 7.32 (d, *J* = 8.0 Hz, 1H), 7.28 (s, 1H), 7.24 (m, 2H), 7.04 (d, *J* = 6.7 Hz, 2H), 7.00 (d, *J* = 7.1 Hz, 2H), 6.73 (d, *J* = 8.0 Hz, 1H), 6.71 (d, *J* = 8.1 Hz, 1H), 5.89 (s, 2H), 3.34 (s broad, 2H), 3.20 (t, *J* = 6.1 Hz, 2H), 2.56 (m, 12H), 1.84 (m, 4H), 1.07 (t, *J* = 7.2 Hz, 6H), 1.03 (t, *J* = 6.8 Hz, 6H). ^{13}C NMR (101 MHz, $CDCl_3$) δ 147.48, 144.78, 136.05, 135.09, 129.05, 128.22, 127.09, 126.05, 123.67, 120.19, 119.80, 118.75, 117.30, 115.71, 113.64, 101.58, 100.80, 58.85, 52.27, 52.09, 46.90, 46.77, 44.37, 43.73, 26.43, 25.61, 11.81, 11.69.

NOE difference experiments

The NMR spectra of **3a**, **3b** and **3c** showed singlets, integrating to three protons at δ_H 3.82, 4.28 or 4.23 ppm respectively, which were attributed to the resonance of N5-CH_3 protons, corroborating N5 alkylation. The NOE ^{1}H-^{1}H spectrum of **3a** showed connectivities between singlet at δ_H 4.28 ppm (N5-CH_3) and the signal corresponding to the proton in position C6 (δ_H 9.53 ppm), which in turn showed connectivity with the singlet of the proton in C7 (δ_H 8.34 ppm, Figure S2). Additionally, this proton resonating at δ_H 8.39 ppm showed long range correlation with the carbon at δ_c 128.94 ppm (C9) in the ^{1}H-^{13}C HMBC spectra, which in turn showed a correlation with the signal at δ_H 7.58 ppm in ^{1}H-^{13}C HMQC spectra. The correlation spectroscopy (COSY) spectrum showed coupling between H9 (δ_H 7.58 ppm) and the signal at δ_H 7.74 ppm, which was then assigned to H10. Additionally, this showed a long-range correlation with two quaternary carbons, resonating at δ_C 114.82 ppm (C6b) and 113.25 ppm (C8), confirming the presence of bromine in the position 8. Similarly, the analysis of the NMR spectra of **3c**, showed a NOE between H6 (δ_H 9.39 ppm) and the doublet resonating at δ_H 8.05 ppm (H7), which in turn, showed a COSY coupling with H8 (δ_H 7.39 ppm), corroborating the presence of a bromine in the position C9. The complete structural elucidation of **3a-c** (Table S2) allowed also to attribute the substitutions pattern in the precursors IQc **1b** and **1c** (NMR characterization in Table S3).

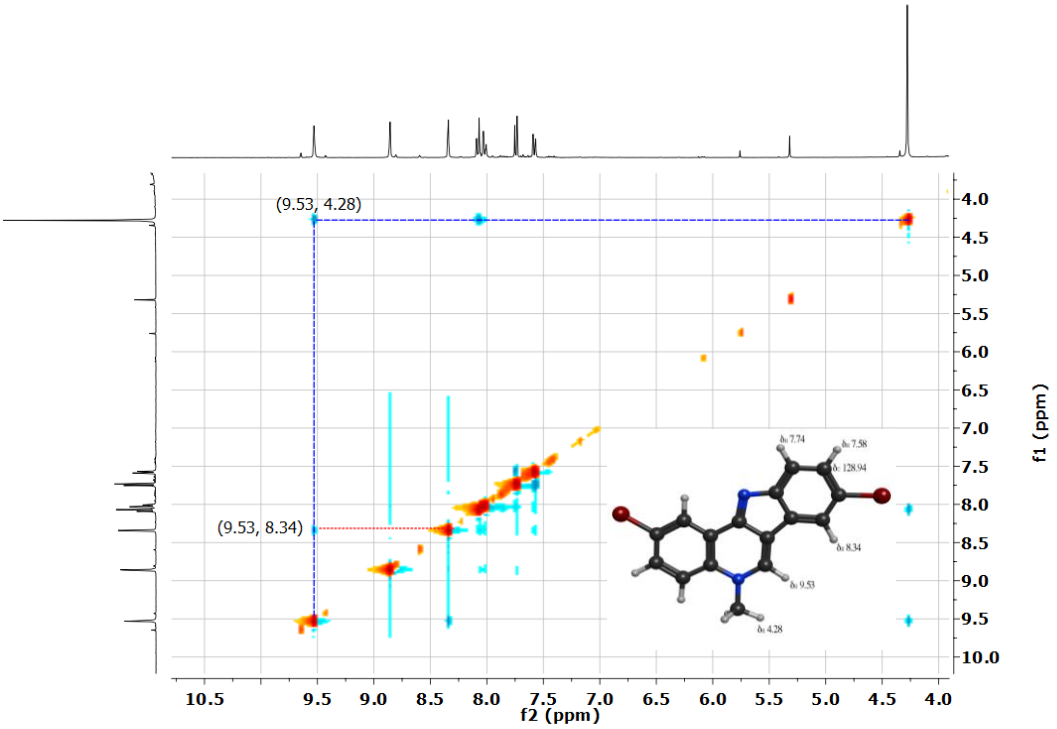

Figure S2 | Nuclear Overhauser effects (NOE) applied to the NMR structural elucidation of **3a** (chemical shifts are in ppm).

NMR chemical shifts assignment of indolo[3,2-*c*]quinoline derivatives

Table S1 –NMR chemical shifts assignment of indolo[3,2-*c*]quinolines **2a, 3a, 3b** and **3c**.

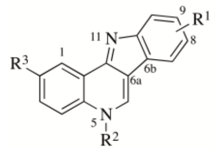

	R^1	R^2	R^3	1a	1	2	3	4	4a	6	6a	6b	7	8	9	10	10a	11a	R^2
				δ_H / ppm<br>δ_C / ppm															
**2a**	H	CH_3	Br	--<br>125.29	8.80<br>126.84	--<br>118.76	7.58<br>131.91	7.95<br>119.02	--<br>133.73	7.78<br>135.19	--<br>121.92	--<br>117.29	7.77<br>119.27	7.31<br>120.84	7.27<br>117.63	7.54<br>126.60	--<br>154.49	--<br>152.06	3.82<br>42.39
**3a**	8-Br	CH_3	Br	--<br>122.28	8.86<br>126.35	--<br>119.29	8.03<br>132.91	8.08<br>121.05	--<br>134.94	9.53<br>140.90	--<br>127.42	--<br>115.82	8.34<br>122.98	--<br>113.25	7.58<br>128.94	7.74<br>120.11	--<br>152.24	--<br>150.56	4.28<br>43.38
**3b**	9-Br	CH_3	Br	--<br>122.86	8.81<br>126.25	--<br>118.91	7.98<br>132.47	8.01<br>120.78	--<br>134.84	9.39<br>139.71	--<br>125.09	--<br>116.52	8.05<br>121.72	7.39<br>123.09	--<br>118.74	7.95<br>121.55	--<br>156.18	--<br>152.82	4.23<br>42.94
**3c**	9-Br	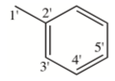	Br	--<br>134.27	8.99<br>127.42	--<br>119.57	7.61<br>132.76	7.43<br>118.85	--<br>133.54	8.57<br>136.59	--<br>120.53	--<br>114.36	7.81<br>120.01	7.58<br>129.78	--<br>126.62	8.01<br>122.38	--<br>152.91	--<br>152.03	5.68(1')<br>58.59(1')<br>133.07(2')<br>7.09(3')<br>126.05(3')<br>7.34 (4')<br>129.42(4')<br>7.34 (5')<br>128.73(5')

Table S2 –NMR chemical shifts assignment of indolo[3,2-*c*]quinolines **1a-c**.

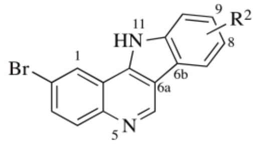

	R^1	1a	1	2	3	4	4a	6	6a	6b	7	8	9	10	10a	11a	N11
		δ_H / ppm<br>δ_C / ppm															
**1a**	H	--<br>118.93	8.81<br>124.93	--<br>118.83	7.86<br>131.32	8.08<br>132.23	--<br>139.28	9.66<br>145.88	--<br>122.05	--<br>115.31	8.34<br>120.79	7.34<br>121.30	7.52<br>126.42	7.74<br>112.48	--<br>139.12	--<br>144.41	12.82<br>--
**1b**	8-Br	--<br>119.07	8.76<br>125.01	--<br>118.81	7.86<br>131.74	8.06<br>132.28	--<br>139.75	9.64<br>146.25	--<br>124.03	--<br>114.54	8.59<br>123.47	--<br>113.59	7.62<br>128.85	7.68<br>114.45	--<br>138.02	--<br>144.65	12.91<br>--
**1c**	9-Br	--<br>119.07	8.80<br>125.03	--<br>118.77	7.86<br>131.62	8.06<br>132.22	--<br>139.56	9.62<br>145.87	--<br>121.20	--<br>114.86	8.28<br>122.54	7.49<br>124.20	--<br>119.01	7.90<br>115.08	--<br>140.16	--<br>144.52	13.00<br>--

Table S3–NMR chemical shifts assignment of indolo[3,2-*c*]quinolines **2b-g** and **3d-j**.

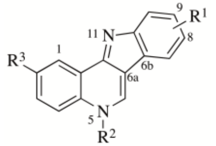

δ_H / ppm
δ_C / ppm

	R^1	R^2	R^3	1a	1	2	3	4	4a	6	6a	6b	7	8	9	10	10a	11a	R2
**2b**	H	CH_3	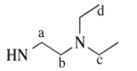	--<br>125.21	7.74<br>103.14	--<br>147.14	6.86<br>119.36	7.22<br>117.87	--<br>128.63	7.99<br>134.63	--<br>122.55	--<br>115.62	7.89<br>118.17	7.42<br>126.43	7.17<br>120.73	7.73<br>119.61	--<br>n.d	--<br>n.d	3.82<br>43.36
**2c**	H	CH_3	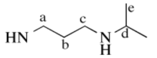	--<br>124.98	7.72<br>102.08	--<br>146.44	6.83<br>118.40	7.21<br>117.20	--<br>127.97	7.95<br>133.74	--<br>122.37	--<br>115.27	7.93<br>118.03	7.45<br>125.73	7.19<br>119.99	7.76<br>118.93	--<br>152.21	--<br>151.65	3.80<br>42.54
**2d**	H	CH_3	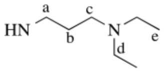	--<br>125.04	7.78<br>102.18	--<br>146.69	6.85<br>118.34	7.26<br>117.22	--<br>127.95	8.02<br>133.79	--<br>122.55	--<br>115.33	7.94<br>118.07	7.45<br>125.69	7.20<br>119.96	7.77<br>118.94	--<br>152.45	--<br>151.67	3.83<br>42.55
**2e**	H	CH_3	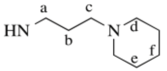	--<br>123.32	7.53<br>101.06	--<br>147.32	6.82<br>119.58	7.29<br>117.57	--<br>127.78	8.31<br>134.78	--<br>120.50	--<br>114.06	7.77<br>115.89	7.37<br>126.37	7.16<br>120.93	7.75<br>119.31	--<br>n.d	--<br>n.d	4.03<br>43.35
**2f**	H	CH_3	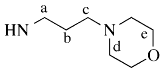	--<br>125.88	7.80<br>102.91	--<br>146.93	6.87<br>118.89	7.26<br>117.80	--<br>128.66	7.97<br>134.23	--<br>123.42	--<br>116.21	7.96<br>119.04	7.47<br>126.25	7.23<br>120.47	7.78<br>119.47	--<br>154.14	--<br>153.01	3.81<br>43.12
**2g**	H	CH_3	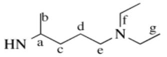	--<br>124.90	7.86<br>103.01	--<br>145.84	6.86<br>118.41	7.30<br>117.48	--<br>127.90	8.15<br>134.11	--<br>122.46	--<br>115.25	7.91<br>117.86	7.42<br>125.75	7.18<br>120.11	7.79<br>119.01	--<br>152.05	--<br>151.04	3.90<br>42.60
**3d**	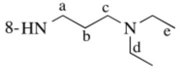	CH_3	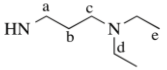	--<br>125.59	7.76<br>102.05	--<br>147.50	6.86<br>120.11	7.36<br>117.98	--<br>128.37	8.23<br>133.78	--<br>122.46	--<br>115.65	7.02<br>99.23	--<br>149.33	6.50<br>110.48	7.55<br>120.44	--<br>152.17	--<br>151.95	4.09<br>43.99
**3e**	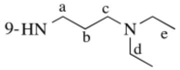	CH_3	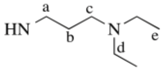	--<br>124.09	7.64<br>102.23	--<br>147.22	6.79<br>119.54	7.22<br>117.84	--<br>128.33	7.94<br>133.07	--<br>121.25	--<br>115.95	7.45<br>120.30	6.50<br>110.20	--<br>149.18	7.00<br>97.64	--<br>152.96	--<br>151.47	4.04<br>43.67
**3f**	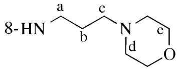	CH_3	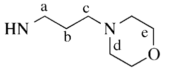	--<br>123.89	7.83<br>101.95	--<br>146.69	6.92<br>120.20	7.36<br>117.74	--<br>128.69	8.05<br>132.64	--<br>121.75	--<br>115.51	7.14<br>101.56	--<br>148.90	6.50<br>110.00	7.60<br>119,40	--<br>151.76	--<br>151.32	4.09<br>43.22
**3g**	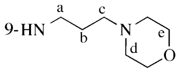	CH_3	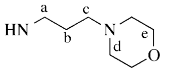	--<br>125.17	7.78<br>102.14	--<br>146.34	6.87<br>118.54	7.35<br>117.08	--<br>128.02	8.10<br>133.87	--<br>121.80	--<br>114.75	7.56<br>119.59	6.55<br>109.21	--<br>148.22	7.07<br>101.24	--<br>153.93	--<br>150.46	4.04<br>42.50
**3h**	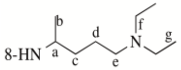	CH_3	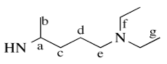	--<br>124.00	7.84<br>103.51	--<br>146.48	6.90<br>119.36	7.37<br>117.94	--<br>128.37	8.09<br>132.96	--<br>122.11	--<br>115.34	7.09<br>100.05	--<br>147.94	6.53<br>110.44	7.58<br>120.34	--<br>152.59	--<br>151.94	4.13<br>43.50
**3i**	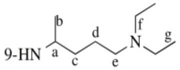	CH_3	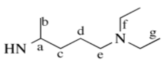	--<br>124.33	7.84<br>102.60	--<br>146.51	6.86<br>118.45	7.34<br>116.23	--<br>128.16	8.00<br>133.16	--<br>121.95	--<br>115.31	7.53<br>120.02	6.52<br>109.96	--<br>148.19	7.11<br>99.45	--<br>153.09	--<br>152.06	4.12<br>44.12
**3j**	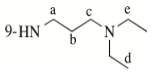	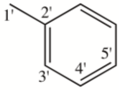	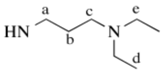	--<br>123.67	7.78<br>101.58	--<br>144.78	6.71<br>117.30	7.60<br>115.71	--<br>127.09	8.86<br>136.05	--<br>120.19	--<br>113.64	7.32<br>128.22	6.73<br>119.80	--<br>147.48	7.28<br>118.75	--<br>n.d	--<br>n.d	5.89(1′)<br>58.85(1′)<br>135.09(2′)<br>7.04(3′)<br>126.05(3′)<br>7.24(4′)<br>129.05(4′)<br>7.00(5′)<br>100.80(5′)

Table S3 (Cont.) - NMR chemical shifts assignment of indolo[3,2-*c*]quinolines **2b-g** and **3d-j** side chains.

				δ_H / ppm<br>δ_C / ppm						
	**R¹**	**R²**	**R³**	**a**	**b**	**c**	**d**	**e**	**f**	**g**
**2b**	H	CH_3	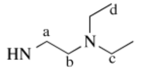	3.24<br>41.75	2.73<br>51.93	2.59<br>47.22	1.06<br>12.44	--	--	--
**2c**	H	CH_3	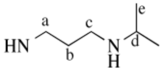	3.30<br>43.13	1.86<br>29.47	2.77<br>45.79	2.83<br>48.89	1.10<br>22.97	--	--
**2d**	H	CH_3	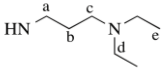	3.32<br>43.91	1.85<br>25.92	2.58<br>52.24	2.58<br>46.92	1.08<br>11.84	--	--
**2e**	H	CH_3	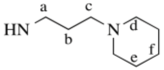	3.26<br>43.65	1.86<br>26.37	2.50<br>58.15	2.46<br>54.82	1.66<br>25.40	1.51<br>24.61	--
**2f**	H	CH_3	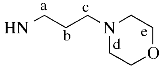	3.33<br>44.12	1.86<br>26.01	2.52<br>58.24	2.50<br>54.47	3.76<br>67.75	--	--
**2g**	H	CH_3	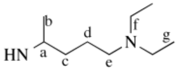	3.72<br>48.46	1.25<br>20.65	1.59<br>35.04	1.59<br>23.72	2.42<br>52.76	2.53<br>46.74	1.02<br>11.51
**3d**	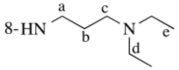	CH_3	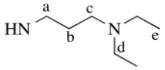	3.28<br>3.36<br>44.63<br>44.70	1.85<br>26.50<br>26.50	2.58<br>52.84<br>53.01	2.58<br>47.58<br>47.61	1.08<br>12.60<br>12.63	--	--
**3e**	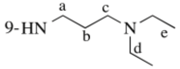	CH_3	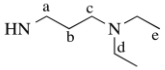	3.37<br>3.39<br>44.45<br>44.74	1.99<br>26.62<br>26.91	2.69<br>52.80<br>52.87	2.69<br>47.55<br>47.60	1.24<br>12.55<br>12.62	--	--
**3f**	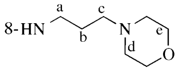	CH_3	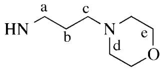	3.45<br>44.11<br>44.44	2.03<br>26.03<br>26.25	2.64<br>58.23<br>58.38	2.64<br>54.48<br>54.50	3.85<br>67.73<br>67.78	--	--
**3g**	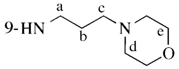	CH_3	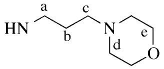	3.31<br>43.26<br>43.34	1.87<br>25.29<br>25.49	2.51<br>57.51<br>57.72	2.51<br>53.77<br>53.80	3.74<br>67.07<br>67.10	--	--
**3h**	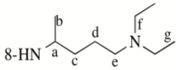	CH_3	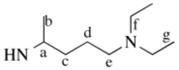	3.86<br>3.73<br>49.19<br>49.53	1.43<br>21.45<br>21.54	1.75<br>35.77<br>35.82	1.75<br>24.41<br>24.45	2.58<br>53.67	2.66<br>47.42<br>47.46	1.20<br>12.26<br>12.34
**3i**	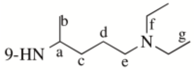	CH_3	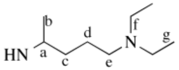	3.75<br>3.86<br>49.18<br>50.10	1.44<br>21.38<br>21.62	1.76<br>35.59<br>35.84	1.76<br>24.42<br>24.52	2.57<br>53.68<br>53.72	2.67<br>47.40<br>47.44	1.20<br>12.11<br>12.26
**3j**	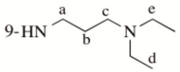	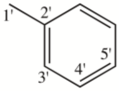	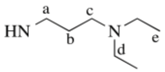	3.34<br>3.20<br>43.73<br>44.37	1,84<br>25.61<br>26.43	2.56<br>52.09<br>52.27	2.56<br>46.77<br>46.90	1.03<br>1.07<br>11.69<br>11.81	--	--

Elemental analysis assays

Table S2 | Predicted and experimental elemental analysis (C,H,N) of indolo[3,2-*c*]quinoline derivatives **2b-g** and **3d-j**.

	Molecular Formula	Predicted	Found
**2b**	$C_{22}H_{27}ClN_4\cdot 2HCl\cdot 1.7H_2O$	C, 54.32; H, 6.71; N, 11.52	C, 54.28; H,7.05; N,11.43
**2c**	$C_{22}H_{27}ClN_4\cdot 2HCl\cdot 2.7H_2O$	C, 52.38; H, 6.87; N, 11.11	C, 52.17; H, 6.51; N, 10.88
**2d**	$C_{23}H_{29}ClN_4\cdot 2HCl\cdot 2.2H_2O$	C, 54.22; H, 7.00; N, 11.00	C, 54.09; H, 7.33; N, 11.13
**2e**	$C_{24}H_{29}ClN_4\cdot 1.3HCl\cdot 4H_2O$	C, 54.55; H, 7.31; N, 10.60	C, 54.54; H, 6.98; N, 10.35
**2f**	$C_{23}H_{27}ClN_4O\cdot HCl\cdot 2.5H_2O$	C, 56.10; H, 6.75; N, 11.38	C, 56.26; H, 6.89; N, 10.90
**2g**	$C_{25}H_{33}ClN_4\cdot 1.9HCl\cdot 2.5H_2O$	C, 55.67; H, 7.46; N, 10.39	C, 55.46; H, 7.06; N, 10.67
**3d**	$C_{30}H_{45}ClN_6\cdot 4HCl\cdot 6.8H_2O$	C, 45.41; H, 7.95; N, 10.59	C, 45.31; H, 7.61; N, 10.72
**3e**	$C_{30}H_{45}ClN_6\cdot 4.4HCl\cdot 3.2H_2O$	C, 48.48; H, 7.57; N, 11.31	C, 48.42; H, 7.69; N, 11.65
**3f**	$C_{30}H_{41}ClN_6O_2\cdot 2.2HCl\cdot 2.3H_2O$	C, 53.40; H, 7.14; N, 12.45	C, 53.44; H, 6.85; N, 10.00
**3g**	$C_{30}H_{41}ClN_6O_2\cdot 2.3HCl\cdot 4H_2O$	C, 50.82; H, 7.29; N, 11.85	C, 50.50; H, 6.95; N, 11.77
**3h**	$C_{34}H_{53}ClN_6\cdot 4HCl\cdot 4H_2O$	C, 51.10; H, 8.20; N, 10.52	C, 50.82; H, 7.89; N, 10.33
**3i**	$C_{34}H_{53}ClN_6\cdot 4HCl\cdot 5.2H_2O$	C, 49.75; H, 8.28; N, 10.24	C, 49.40; H, 7.93; N, 9.93
**3j**	$C_{36}H_{49}ClN_6\cdot 4HCl\cdot 2.5H_2O$	C, 54.58; H, 7.38; N, 10.61	C, 54.32; H, 7.51; N, 10.95

FRET melting assays

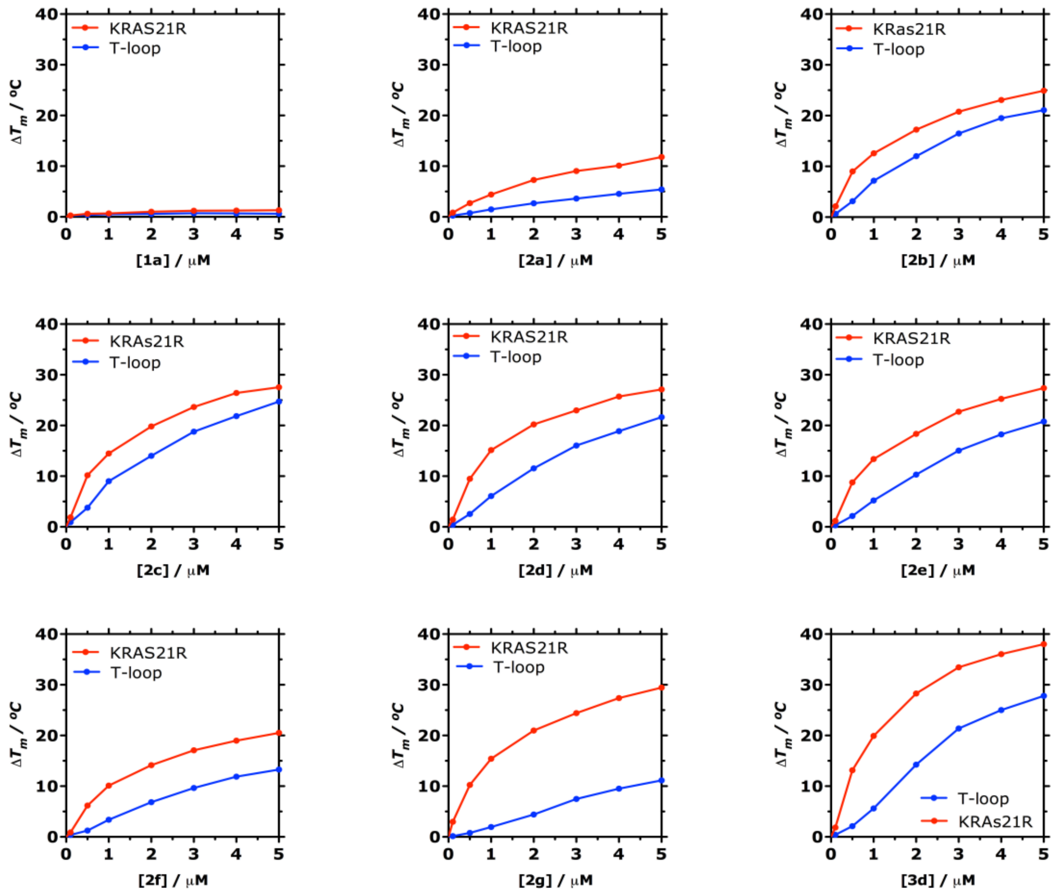

Figure S3 | Concentration-dependent (0.1, 0.5, 1, 2, 3, 4 and 5 μM) FRET melting profiles of G4 structures and T-loop DNA with indolo[3,2-*c*]quinoline derivatives **1a**, **2a-g** and **3d-j** in K-cacodylate buffer (pH 7.4, 60 mM K^+).

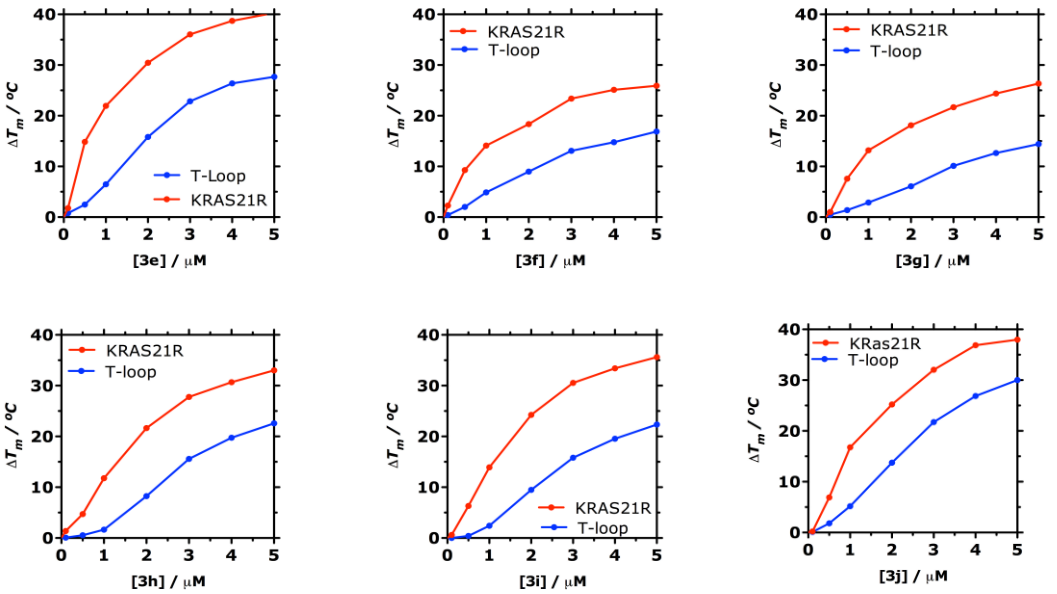

Figure S3 (cont.)| Concentration-dependent (0.1, 0.5, 1, 2, 3, 4 and 5 μM) FRET melting profiles of G4 structures and T-loop DNA with indolo[3,2-*c*]quinoline derivatives **1a**, **2a-g** and **3d-j** in K-cacodylate buffer (pH 7.4, 60 mM K^+).

a) b)

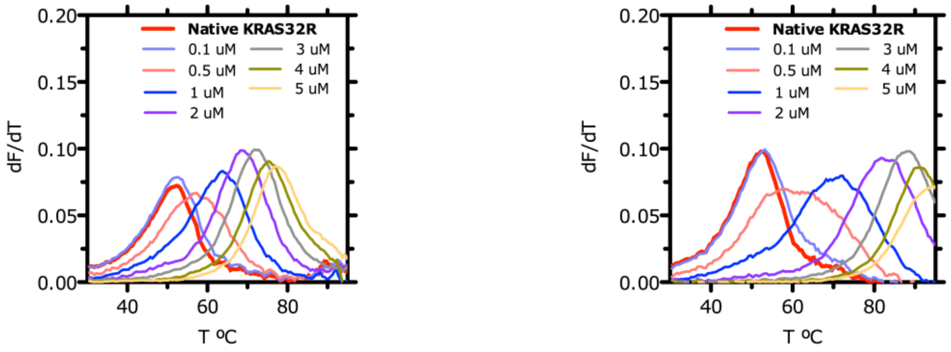

Figure S4 | First derivative of the FRET data (dF/dT) for the stabilization of the KRAS32R G4 with a) **2d** and b) **3e** (0.1, 0.5, 1, 2, 3, 4 and 5 μM) in in K-cacodylate buffer (pH 7.4, 60 mM K^+)

Table S5 | FRET stabilization temperatures (ΔT_m) of G4s sequences and ds-DNA (0.2 μM) stabilized by IQc derivatives (1 μM).

	R¹	R²	ΔT_m (ºC)[a]				R¹	R²	ΔT_m (ºC)[a]	
			**KRas21R**	**T-loop**					**KRas21R**	**T-loop**
**1a**	Br	--	0.7	0.4	**3c**		Br	CH_2Ph	2.7	0.3
**2a**	Br	CH_3	4.4	1.5	**3d**	2,8	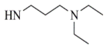	CH_3	19.9	5.6
**2b**	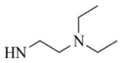	CH_3	12.6	7.2	**3e**	2,9	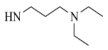	CH_3	22.0	6.5
**2c**	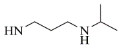	CH_3	14.5	9.1	**3f**	2,8	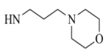	CH_3	14.1	4.9
**2d**	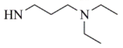	CH_3	15.1	6.1	**3g**	2,9	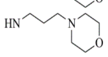	CH_3	13.2	2.9
**2e**	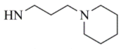	CH_3	13.4	5.2	**3h**	2,8	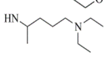	CH_3	11.8	1.7
**2f**	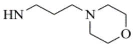	CH_3	10.1	3.4	**3i**	2,9	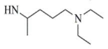	CH_3	13.9	2.4
**2g**	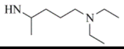	CH_3	15.4	1.9	**3j**	2,8	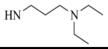	CH_2Ph	16.8	5.2

[a] SD ≤ 0.2 ºC; T_m (KRas21R) = 52.1±0.1 ºC; T_m (T-loop) = 53.6±0.1 ºC.

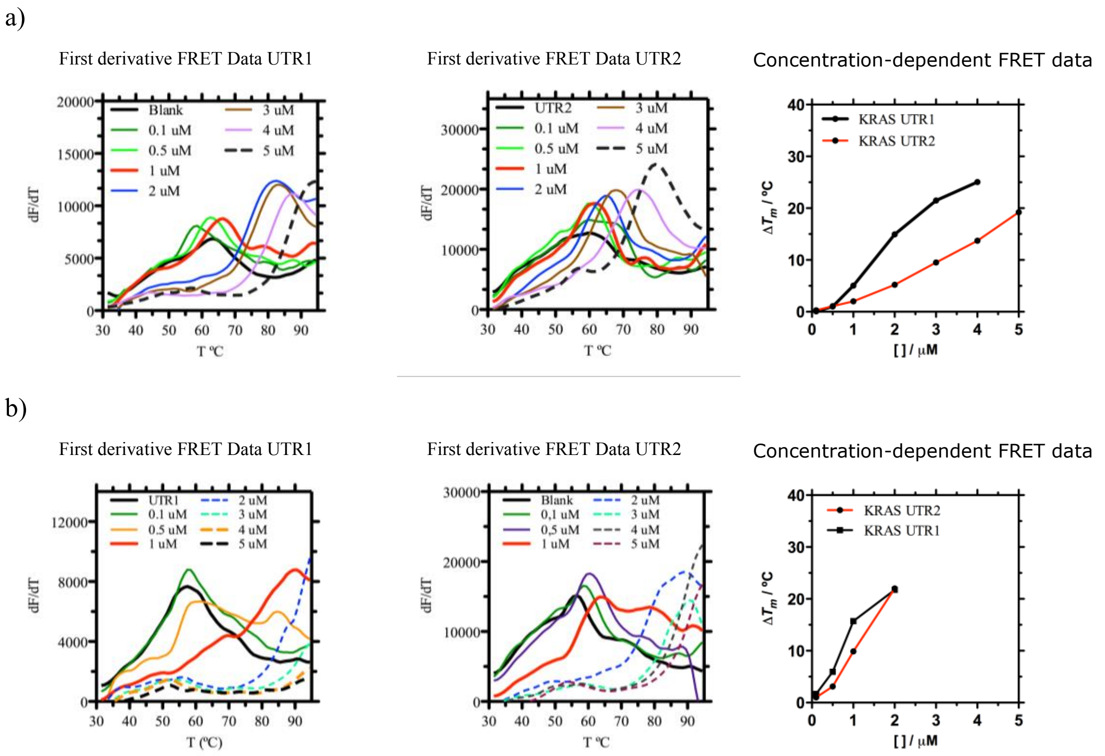

Figure S5. First derivative of the FRET data (dF/dT) for the stabilization of the KRAS UTR1, UTR2 G4 and concentration-dependent FRET melting profiles with a) 2d and b) 3e (0.1, 0.5, 1, 2, 3, 4 and 5 μM) in in K-cacodylate buffer (pH 7.4, 60 mM K^+)

Fluorescence spectra of saturation binding studies

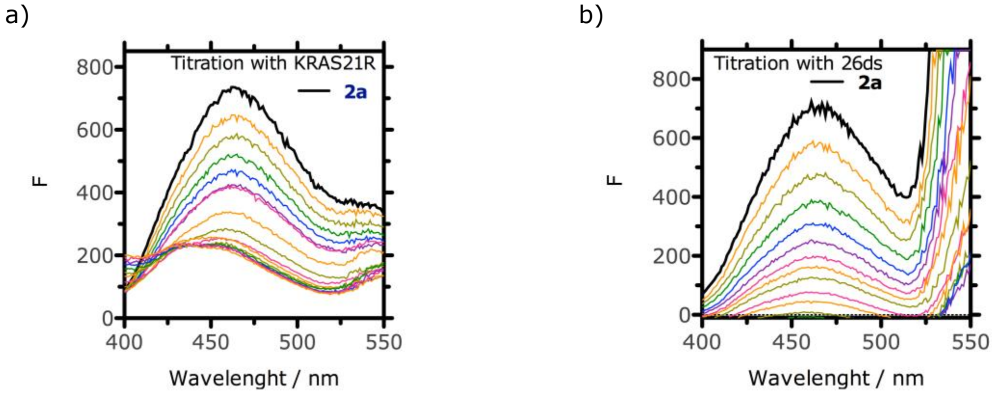

Figure S6. Fluorescence titrations of 1 μM indolo[3,2-*c*]quinoline **2a** (1μM) with a) KRAS21R G4 and b) 26ds DNA in K-cacodylate buffer pH 7.4, containing 60 mM K^+ at 25 ºC. Concentration of DNA increase from the top to the bottom (grey lines) Excitation wavelength of 275 nm.

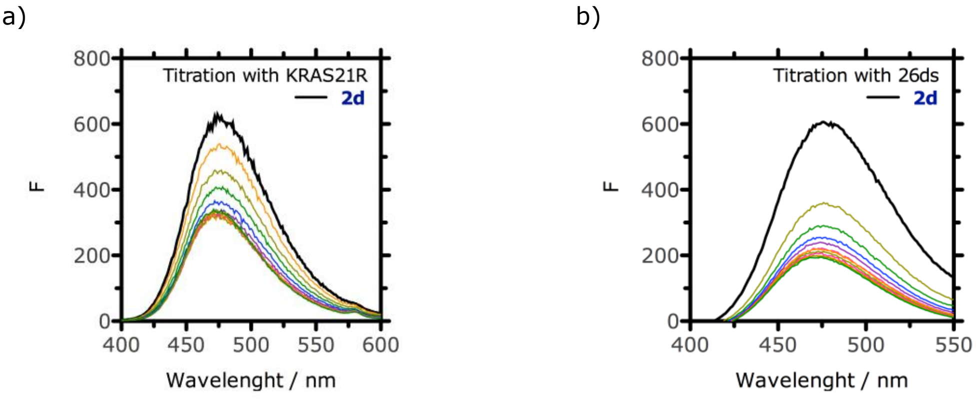

Figure S7. Fluorescence titrations of 1 μM indolo[3,2-*c*]quinoline **2d** (1μM) with KRAS21R G4 and 26ds DNA in K-cacodylate buffer pH 7.4, containing 60 mM K^+ at 25 ºC. Concentration of DNA increase from the top to the bottom (grey lines) Excitation wavelength of 290 nm.

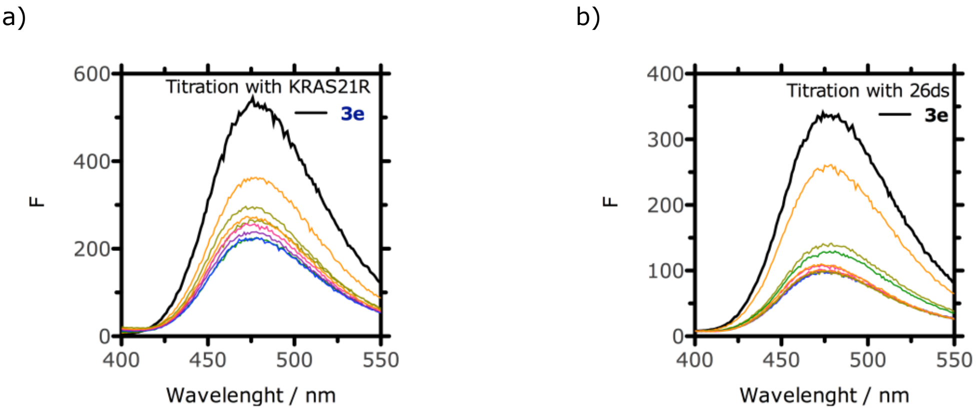

Figure S8. Fluorescence titrations of 1 μM indolo[3,2-*c*]quinoline **3e** (1μM) with KRAS21R G4 and 26ds DNA in K-cacodylate buffer pH 7.4, containing 60 mM K^+ at 25 ºC. Concentration of DNA increase from the top to the bottom (grey lines) Excitation wavelength of 290 nm.

The titration data obtained were used to construct Scatchard plots with the concentration of bound and free ligand (C_b and C_f, respectively). From these data, the values of *r*, the number of moles of ligand bound to 1 mol of G4 ($r = C_b/C_{DNA}$) were calculated.

Saturation binding isotherms were represented as Scatchard plots, by plotting r/C_f *versus* *r* and evaluated according to the model of McGhee and von Hippel. Dissociation constants were determined by fitting the titration experimental data to one-site saturation binding equation or to one-site saturation binding to the Hill slope equation supplied with the GraphPad PRISM software (GraphPad, Version 5.00, San Diego, CA).

Association constants (K_a) were calculated from dissociation constants (K_d). Scatchard lines represented in the Scatchard plots were calculated from the data fitting to the binding equations ($x = 0$, $y = F_{max}/K_d$; $x = F_{max}$, $y = 0$; slope = $-K_a$).

Circular dichroism assay

a) b)

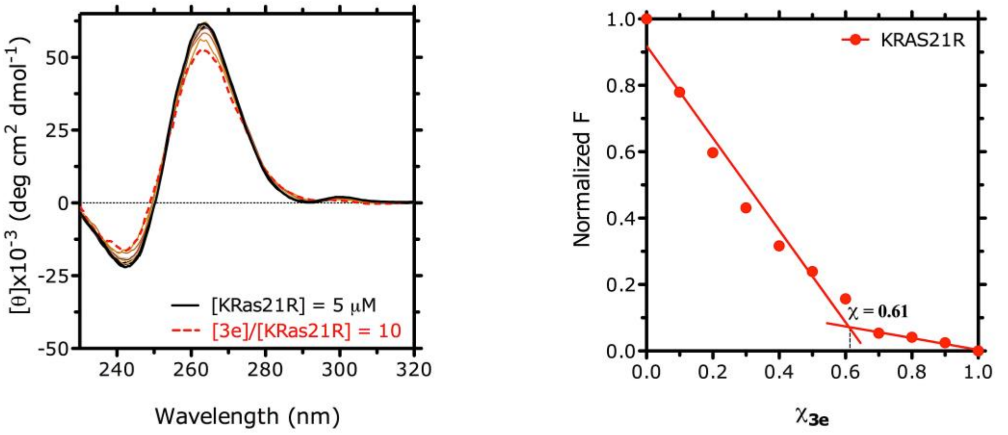

Figure S9 | a) CD titration spectra of the KRAS21R and induced by titration with **3e** in K-cacodylate buffer pH 7.4, containing 60 mM K^+ at 25 ºC. Solid black line – Pure G4 DNA at 5 uM. Ligand:G4 complex ratios were: 0, 0.5, 1.0, 1.5, 2.0, 2.5, 3.0, 5.0, 7.5, 10.0, sequentially from the top around 260 nm; b) Job Plot of **3e** in complex with KRAS21R G4 in K-cacodylate buffer pH 7.4, containing 60 mM K^+, at 25 ºC. The sum of the concentrations of **3e** and G4 was kept constant at 4 μM.

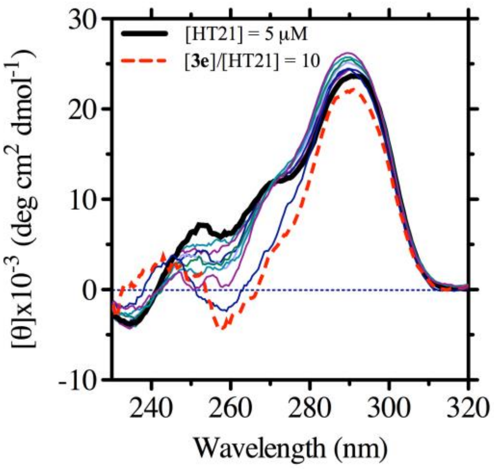

Figure S10. CD titration spectra of HT21 G4 (5 μM) in K^+ cacodylate buffer (pH 7.4, containing 60 mM K^+), at 25 ºC (solid black line) and titrations with IQc 3e. Ligand:G4: ratios were: 0, 0.5, 1.0, 1.5, 2.0, 2.5, 3.0, 5.0, 7.5, 10.0, sequentially from the top around 255 nm.

The native folding topology of the HT21 G4 exhibited a typical CD spectrum of a parallel/antiparallel hybrid topology (solid black line). This is a positive band having a maximum around 290 nm, a shoulder (+) centred around 270 nm, a weak band (+) with a maximum around 255 nm and a negative band with a maximum at 235 nm.[4]

The titration of HT21 with **3e** caused a gradual suppression of the shoulder at 270 nm; the weak positive band at 255 nm became a weak negative band with a maximum at ca. 260 nm (for a **3e**:HT21 ratio of 10); and an isodichroic point arose at 270 nm. All these observations suggest that the IQc derivative induces a conformational change to an anti-parallel G4 topology

Sulforhodamine B (SRB) short-term cytotoxicity assay

Cells were seeded at appropriate densities into the wells of 96 well plates in their corresponding medium and incubated overnight to allow the cells to attach. Subsequently cells were exposed to various concentrations of freshly made solutions of drugs and incubated for 96 h. The cells were subsequently fixed with ice-cold trichloacetic acid (TCA) (10%, w/v) for 30 min and stained with 0.4% SRB dissolved in 1% acetic acid for 15 min. All incubations were carried out at room temperature except for TCA fixation, which was at 4 °C. The IC_{50} value, the concentration required to inhibit cell growth by 50%, was determined from the mean absorbance at 540 nm for each drug concentration expressed as a percentage of the control untreated well absorbance. (Performed at School of Pharmacy, University College of London).

Total protein extraction and immunoblotting

Samples were homogenized in ice-cold 1:1 solution of buffer A [10 mm Tris•HCl pH 7.6, 5 mm MgCl2, 1.5 mm KOAc, 2 mm dithiothreitol (DTT), and Halt Protease and Phosphatase inhibitor cocktail, EDTA-free (#78445, Thermo Scientific)] and buffer 2X (10 mm Tris•HCl pH 7.6, 1% Nonidet-P40, and Halt Protease and Phosphatase inhibitor cocktail), by vigorous vortexing and incubated on ice for 30 min. Samples were then sonicated (two cycles of 15 s sonication and 30 s ice incubation, using a compact ultrasonic device with amplitude adjusted to 80% and pulse to 90%; model UP100H, Hielscher Ultrasonics GmbH, Teltow (Germany); 100 W, ultrasonic frequency: 30 kHz) and centrifuged at 10000 g for 10 min at 4ºC. The clear supernatants containing the total protein extracts were transferred to a fresh tube and stored at −80ºC. Protein concentrations were determined using the BioRad protein assay kit according to the manufacturer's instructions. Steady-state levels of KRAS protein were determined by immunoblot analysis. Briefly, 50-100 µg of total protein extracts were separated by 12% SDS-PAGE. After electrophoretic transfer onto nitrocellulose membranes, immunoblots were blocked with 5% milk solution, and next incubated overnight at 4ºC with primary mouse monoclonal antibody reactive to KRAS/p53 (#sc-30/sc-126; Santa Cruz Biotechnology Inc., Santa Cruz, CA, USA). Finally, immunoblots were incubated with secondary anti-mouse antibody conjugated with horseradish peroxidase (BioRad) for 3 h at room temperature. The membranes were processed for protein detection using Super Signal substrate (Pierce, Rockford, IL, USA). β-Actin (#A-5441, Sigma–Aldrich) was used as a loading control. Steady-state protein levels were expressed as mean ±SEM from at least three independent experiments.

Total RNA extraction and Taqman real-time RT-PCR

Taqman Real-time PCR reactions were performed using 1.5 μg of total RNA, using High Capacity RNA to cDNA kit, according to the manufacturer`s instruction. Next, Real-time PCR reactions were performed using Taqman Universal Master Mix II, no UNG, and primers specific to human KRAS (assay ID Hs00000174_rf, # 4465807) primers and to Human β-Actin (ACTB # 401846) for normalization to endogenous control (both from Applied Biosystems Inc). Triplicate reactions were run per sample. Data was collected with 7300 System Sequence Detection Software, version 1.2.3 (Applied Biosystems Inc). The comparative threshold cycle method was used to calculate the amplification factor, where the threshold cycle (Ct) is defined as the cycle number at which the fluorescence passes the fixed threshold intensity level. KRAS expression levels in different samples were calculated on the basis of ΔΔCt method. For each cell line, vehicle control (DMSO) was used as the calibrator. The n-fold change in KRAS expression was obtained using the formula: 2-ΔΔCt.

Guava ViaCount assay

The ViaCount Assay distinguishes viable mid apoptotic and dead cells based on differential permeability of two DNA-binding dyes in the Guava ViaCount Reagent. The nuclear dye stains only nucleated cells, while the viability dye brightly stains dying cells. HCT116, SW620 and HEK293 T cells were seeded in 24-well plates 50,000cells/well. Twenty-four hours later, cells were exposed to compounds for 72 h. After treatment, cell culture supernatants were collected and adherent cells were detached with TrypLE (Invitrogen). Next, detached cells were pooled with cell culture supernatants and centrifuged for 5 min (650 g). Supernatants were discarded and the cells were resuspended in 50-500μl phosphate buffered saline (PBS) with 2% FBS. Subsequently, 15 μl of cell suspension were mixed with 135 μl of Guava ViaCount reagent, and incubated for 5 min at room temperature. Sample acquisition and data analysis were performed using the ViaCount software module.

Hoechst Staining

Hoechst labeling of cells was used to detect apoptotic nuclei by evaluation of nuclear morphology under fluorescence microscopy. In brief, cells were fixed in 4% paraformaldehyde in phosphate-buffered saline (PBS), pH 7.4, for 10 min at 25 °C, incubated with Hoechst dye 33258 (Sigma Chemical Co.) at 5 mg/mL in PBS for 5 min, washed with PBS, and mounted using PBS/glycerol (3:1, v/v). Fluorescent nuclei were scored and categorized according to the condensation and staining characteristics of chromatin. Normal nuclei showed noncondensed chromatin dispersed over the entire nucleus. Apoptotic nuclei were identified by condensed chromatin, contiguous to the nuclear membrane, as well as nuclear fragmentation of condensed chromatin. Three random microscopic fields per sample of approximately 100 nuclei were observed.

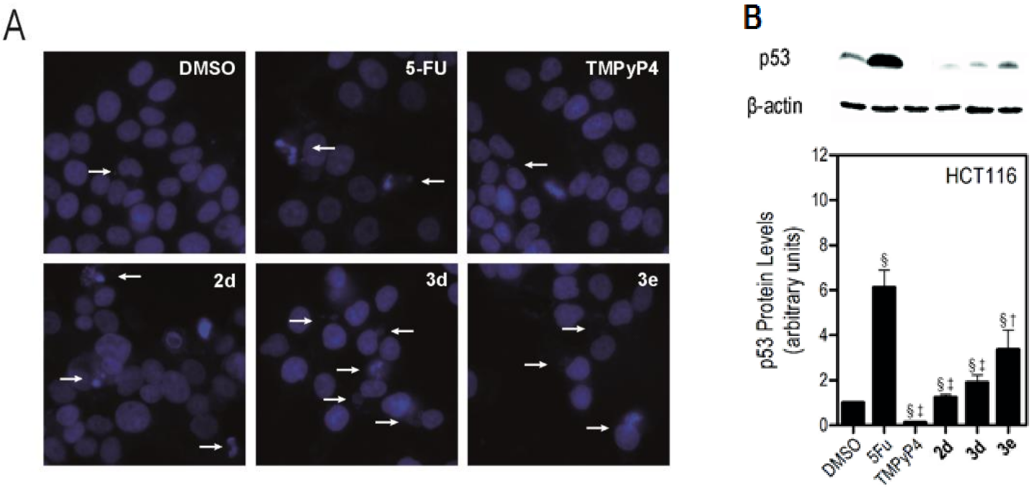

Figure S11. Exposure to IQc compounds increases apoptosis and p53 protein expression in HCT116 cells. **A.** Nuclear morphology and representative images of Hoechst staining of HCT116 cells after Hoechst staining, evaluated by fluorescence microscopy after 72 h exposure to equitoxic (IC_{50}) concentrations of 5-FU, TMPyP4 and IQc (**2d, 3d, 3e**) treatment or DMSO (vehicle control) at 400x magnification. Arrows indicate nuclear fragmentation and chromatin condensation, and: **B.** p53 protein steady-state expression evaluated by immunoblot relative to DMSO (vehicle control), after 72 h exposure of HCT116 cells to equitoxic (IC_{50}) concentrations of 5-FU, TMPyP4 and IQc. Results are expressed as mean ±SEM of at least three independent experiments; *p < 0.05 and §p < 0.01 from DMSO (vehicle control); and †p <0.05 and ‡p < 0.01 from 5-FU.

References

1 Gould, R. G. & Jacobs, W. A. The Synthesis of Certain Substituted Quinolines and 5,6-Benzoquinolines. *J. Am. Chem. Soc.* **61**, 2890–2895, doi:10.1021/ja01265a088 (1939).

2 Molina, A. *et al.* Synthesis and DNA binding properties of gamma-carbolinium derivatives and benzologues. *J. Org. Chem.* **61**, 5587-5599 (1996).

3 Pereira, C. M. P., Stefani, H. A., Guzen, K. P. & Orfao, A. T. G. Improved synthesis of benzotriazoles and 1-acylbenzotriazoles by ultrasound irradiation. *Lett. Org. Chem.* **4**, 43-46 (2007).

4 Dai, J., Carver, M. & Yang, D. Polymorphism of human telomeric quadruplex structures. *Biochimie* **90**, 1172-1183, doi:10.1016/j.biochi.2008.02.026 (2008).
